# The origin of different bending stiffness between double-stranded RNA and DNA revealed by magnetic tweezers and simulations

**DOI:** 10.1093/nar/gkae063

**Published:** 2024-02-07

**Authors:** Hai-Long Dong, Chen Zhang, Liang Dai, Yan Zhang, Xing-Hua Zhang, Zhi-Jie Tan

**Affiliations:** School of Physics and Technology, College of Life Sciences, Renmin Hospital of Wuhan University, Wuhan University, Wuhan 430072, China; School of Physics and Technology, College of Life Sciences, Renmin Hospital of Wuhan University, Wuhan University, Wuhan 430072, China; Department of Physics, City University of Hong Kong, Hong Kong 999077, China; Department of Clinical Laboratory, Renmin Hospital of Wuhan University, Wuhan 430072, China; School of Physics and Technology, College of Life Sciences, Renmin Hospital of Wuhan University, Wuhan University, Wuhan 430072, China; School of Physics and Technology, College of Life Sciences, Renmin Hospital of Wuhan University, Wuhan University, Wuhan 430072, China

## Abstract

The subtle differences in the chemical structures of double-stranded (ds) RNA and DNA lead to significant variations in their biological roles and medical implications, largely due to their distinct biophysical properties, such as bending stiffness. Although it is well known that A-form dsRNA is stiffer than B-form dsDNA under physiological salt conditions, the underlying cause of this difference remains unclear. In this study, we employ high-precision magnetic-tweezer experiments along with molecular dynamics simulations and reveal that the relative bending stiffness between dsRNA and dsDNA is primarily determined by the structure- and salt-concentration-dependent ion distribution around their helical structures. At near-physiological salt conditions, dsRNA shows a sparser ion distribution surrounding its phosphate groups compared to dsDNA, causing its greater stiffness. However, at very high monovalent salt concentrations, phosphate groups in both dsRNA and dsDNA become fully neutralized by excess ions, resulting in a similar intrinsic bending persistence length of approximately 39 nm. This similarity in intrinsic bending stiffness of dsRNA and dsDNA is coupled to the analogous fluctuations in their total groove widths and further coupled to the similar fluctuation of base-pair inclination, despite their distinct A-form and B-form helical structures.

## Graphical Abstract

**Graphical Abstract:**
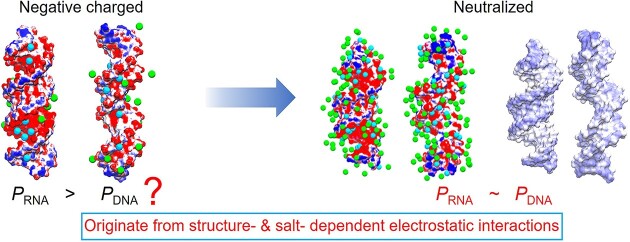

## Introduction

The stiffness or elasticity of double-stranded RNA (dsRNA) and double-stranded DNA (dsDNA) play crucial roles in various biological processes, including structural packaging (1), interactions with proteins (2,3), transcription and translation (2,4), as well as nanostructure designs such as RNA and DNA origami (5). Extensive single-molecule experiments including magnetic tweezers (MT) and optical tweezers and all-atom molecular dynamics (MD) simulations have been conducted to investigate and comprehend the elasticity of dsRNA and dsDNA in different dimensions such as bending, stretching, and torsional elasticity as well as twist-stretch coupling (6–12). Moreover, the influence of ions (13–19), temperature (20,21), and sequences (22–26) on the elasticities of dsRNA and dsDNA have also been extensively investigated by single-molecule experiments and MD simulations.

Compared to B-form dsDNA, A-form dsRNA generally exhibits a visibly larger bending persistence length (*P*) (10,27–29), a significantly smaller stretch modulus (27,29), similar torsional persistence length (27), and opposite twist-stretch coupling (9,27,30) under near-physiological salt conditions. The different bending stiffness of A-form dsRNA and B-form dsDNA has been identified as a key factor contributing to the two-order magnitude of difference in buckling dynamics, such as characteristic transition rate, between dsRNA and dsDNA (27,31–33). The comparatively softer stretching elasticity of A-form dsRNA has been ascribed to its larger base-pair inclination and the resultant more open structure than B-from dsDNA, as shown in recent all-atom MD simulations (34–36). The opposing twist-stretch couplings of dsRNA and dsDNA have been collectively linked to two competing deformation pathways when stretched, as demonstrated in state-of-the-art MD simulations: shrinking helical radius results in negative coupling for dsDNA, while a widening major groove leads to a positive coupling for dsRNA (9,34,35). Moreover, our recent MT experiments and MD simulations revealed that multivalent cations can reverse the twist-stretch coupling of dsRNA through changes in the deformation pathway (9). However, the visibly higher bending stiffness of A-form dsRNA compared to B-form dsDNA remains poorly understood.

Due to the polyanionic nature of dsRNA and dsDNA, their bending persistence lengths are sensitive to ion conditions (7,29,37–40). For instance, recent single-molecule experiments demonstrated that *P*_DNA_ and *P*_RNA_ decrease from ∼55 to ∼44 nm and from ∼67 to ∼53 nm respectively, as NaCl concentration ([NaCl]) increases from ∼0 to ∼500 mM (10). Recent MT experiments and all-atom MD simulations have revealed that beyond the traditional view of monovalent ions' neutralization roles, multivalent ions exhibit seemingly contrasting effects on *P*_RNA_ and *P*_DNA_ (29,41). Nonetheless, high-precision single-molecule studies examining the effects of monovalent salt on the relative bending stiffness of dsRNA and dsDNA have rarely been extended to very high salt concentrations (10,42–44). Consequently, a comprehensive understanding of the relative bending stiffness of A-form dsRNA and B-form dsDNA at very high monovalent salt concentrations also remains elusive.

In this study, we employed a combination of high-precision MT experiments, MD simulations, and Poisson-Boltzmann (PB) calculations to quantify the relative bending stiffness of dsRNA and dsDNA until very high salt concentrations and to investigate the underlying mechanisms of why dsRNA is stiffer than dsDNA at near-physiological salts.

## Materials and methods

### High-precision measurements of the force–extension curves of RNA and DNA by magnetic tweezers

**Figure 1.**
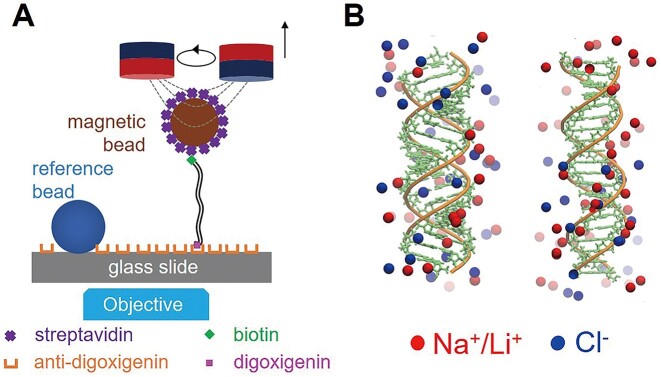
Our MT experiments and MD simulations to determine the elasticities of dsDNA and dsRNA. (**A**) The home-built magnetic tweezer. A pair of NdFeB magnets are used to stretch the molecule anchored between a glass slide and a microbead. (**B**) The initial structures of 20-bp dsRNA (left) and dsDNA (right) used in our MD simulations with added Na^+^/Li^+^ (red) and Cl^−^ (blue) ions.

We conducted all MT measurements in an ultra-clean room to prevent RNA degradation and maintained a constant temperature of 22°C to minimize draft which was mainly caused by temperature fluctuations during the day-long experiments; see Figure 1A. We labeled each DNA or RNA molecule with biotin and a digoxigenin group at either end. Details of the preparation of the RNA and DNA constructs were similar to our previous work (45) but with a different 13 751-bp sequence (Supplementary Figure S1). After attaching polystyrene beads (3 μm diameter) to the glass surface as reference points, we anchored the digoxigenin-labeled ends of the RNA or DNA molecules to the anti-digoxigenin-coated glass surface. Subsequently, we passivated the glass surfaces overnight using 2% bovine serum albumin (Sigma-Aldrich) and 0.1% beta-mercaptoethanol (Sigma-Aldrich) in phosphate-buffered saline (PBS). We then attached superparamagnetic microbeads (Dynabeads MyOne Streptavidin C1) to the biotin-labeled ends of the DNA or RNA molecules in PBS and passivated the surfaces using mPEG-Succinimidyl Valerate ester (MW 350, Hunan Huateng Pharmaceutical) in 100 mM sodium bicarbonate solution. We conducted all MT measurements using buffers containing 1 mM Tris–HCl pH 7.5 supplemented with various concentrations of NaCl or LiCl.

To acquire accurate force-extension curves for DNA and RNA across a broad range of salt concentrations, we implemented several specific strategies in our MT experiments. (i) Correction based on the buffer's refractive index. Since the buffer's refractive index (*n*_b_) influenced the diffraction pattern of the paramagnetic beads, we measured *n*_b_ for each salt concentration using a refractometer (PAL-RI, ATAGO Japan). We then recalibrated the bead's diffraction pattern at each salt concentration using a piezo objective scanner and recalculated the extension for each DNA or RNA molecule based on the measured *n*_b_ (Supplementary Figure S2). (ii) Selection of the paramagnetic beads. We selected to use DNA or RNA molecules anchored to the center bottom of the beads to minimize errors in the measured extension resulting from discrepancies between the axis of the force applied to the bead and the axis of the DNA/RNA molecule (Supplementary Figure S3). (iii) Determining the glass top's height. To obtain the absolute extension (i.e. the height difference between the bottom of the paramagnetic bead and the top of the functionalized glass surface) of the DNA and RNA, we determined the glass top's height at zero force, where the paramagnetic bead touched the glass (Supplementary Figure S4). (iv) Determining the applied force. To determine the exact applied force (*F*) at each magnets' height (*h*), we fitted *F* as a function of *h* for each paramagnetic bead (Supplementary Figure S5). (v) To prevent the interactions between the DNA, the glass, and the beads which may lead to shorter contour lengths, we passivated all surfaces using mPEG-Succinimidyl Valerate ester (MW 2K, Hunan Huateng Pharmaceutical) in 100 mM sodium bicarbonate solution for two hours before the measurement of force-extension curves.

We determined the effects of monovalent salts on the persistence lengths *P*_RNA_ and *P*_DNA_ through measurements of the force-extension (*F–x*) curves. We fitted each *F-x* curve using the following formula (46), which uses a seventh-order polynomial to provide all the correction terms to the residuals of the Marko–Siggia interpolation formula of the worm-like chain (WLC) model (47):


(1) \begin{eqnarray*}F = \frac{{{k}_{\mathrm{B}}T}}{P}\left[ {\frac{1}{{4{{\left( {1 - x/{L}_{\mathrm{c}}} \right)}}^2}} - \frac{1}{4} + \frac{x}{{{L}_{\mathrm{c}}}} + \mathop \sum \limits_{i = 2}^{i \le 7} {\alpha }_i{{\left( {\frac{x}{{{L}_{\mathrm{c}}}}} \right)}}^i} \right].\end{eqnarray*}

Here, *x* and *F* are measured extension and force in MT experiments. To avoid underestimating the experimental data at low force, we used *x* and the logarithm of *F* as variables in fitting arithmetic using the least square method. The fitting parameters, *L_c_* and *P* are the contour length and bending persistence length, respectively (45). Please see the previous work for the polynomial coefficients *α_i_* (46). Our measurements were extended to very high monovalent salt concentrations such as 4 M NaCl and 6 M LiCl. For each salt condition, we repeated measurements on at least ten molecules to obtain average values and standard errors for *L_c_* and *P*.

### All-atom molecular dynamics simulations

To elucidate the underlying mechanisms governing the salt-dependence of persistence length *P*_RNA_ and *P*_DNA_, we conducted all-atom MD simulations with the dsDNA sequence of CGACTCTACGGCATCTGCGC and the corresponding sequence for dsRNA, with thymine (T) bases replaced by uracil (U), based on previous experiments with short DNA fragments (9,29,48,49). We constructed the initial structures of dsRNA and dsDNA using the nucleic acid builder (NAB) of AMBER (Figure 1B) (50). Our simulations were carried out using the Gromacs 4.6 software package with recently refined AMBER ff99bsc1+χOL3 force fields and a TIP3P water model for molecular interactions (50–53). We added Na^+^/Li^+^ and Cl^−^ ions to ensure that our simulated systems were neutralized with the ion model from Joung and Cheatham (54–56).

Based on our MT experiments, we chose typical near-physiological salt conditions (150 mM NaCl, 150 mM LiCl), high salt conditions (1 M NaCl, 1 M LiCl) (55,56), and very high salt conditions (4 M NaCl, 6 M LiCl). In each simulation, the dsRNA or dsDNA was placed in the center of the rectangular box, and the periodic boundary conditions along with the particle mesh Ewald (PME) summation method was employed for the Coulomb interactions and a smooth cut-off of 10 Å was used for the van der Waals energy (57). An integration step of 2 fs was used in conjunction with the leap-frog and LINCS algorithms (58). The systems were energy-minimized for 5000 steps and then were heated to 298 K and equilibrated with the Nosé–Hoover temperature coupling until 100 ps under isothermal-isochoric ensemble (NVT) conditions (59). Afterward, we conducted the 100 ps equilibrations under isothermal–isobaric ensemble (NPT) conditions with Parrinello–Rahman pressure coupling (60). In the 100 ps NVT and NPT simulations, nucleic acids were restrained by a harmonic potential with a force constant of 1000 kJ/mol·nm^−2^. Subsequently, all production MD simulations at very high salt concentrations (4 M NaCl and 6 M LiCl) were performed till 1000 ns, while those at 150 mM and 1 M NaCl/LiCl were conducted till 600 ns in the NPT ensemble (pressure = 1 atm, temperature = 298 K). As shown in Supplementary Figure S7, the dsRNA and dsDNA reach their equilibrium after ∼200 ns, and the last 500- or 400-ns trajectories were used for the analyses at very high salts or other salts, respectively. In addition, our simulated systems were confirmed to be neutralized with the desired salt concentrations, as shown in Supplementary Figure S8.

Furthermore, we performed MD simulations for the electrically ‘neutral’ dsRNA and dsDNA (named dsRNA* and dsDNA*), to examine the intrinsic (non-electrostatic) bending stiffness of dsRNA and dsDNA (61,62). Specifically, we built the electrically ‘neutral’ dsRNA/dsDNA by adding a charge of +1e to each phosphate group, i.e. the atom charges of phosphate groups were reduced in the same proportions to equivalently add a +1e charge to each phosphate group and the charges of other atoms were kept unchanged; see the detailed partial atom charges of phosphate groups in Supplementary Table S3. The MD simulations for the electrically ‘neutral’ dsRNA/dsDNA were performed until 600 ns at 150 mM NaCl according to the procedure described above; see Supplementary Figure S7.

### Calculation of bending persistence length

Based on the probability *p*(*θ*) at bending angle *θ* from the MD trajectories, we calculated *P*_RNA_ and *P*_DNA_ through fitting *p*(*θ*) to the equation (41)


(2) \begin{eqnarray*}- \ln \left( {p\left( \theta \right)/{\mathrm{sin}}\theta } \right) = P{\theta }^2/2{L}_c,\end{eqnarray*}

where *θ* is the bending angle over the contour length *L*_c_. For a convenient comparison, we used the segments with 13-bp and with 11-bp in our calculations for dsRNA and dsDNA, as those segments approximately have the similar contour lengths *L*_c_ of ∼3.3 nm. Please note that the respective *L_c_* values obtained from our MD simulations were utilized in the calculations for persistence length *P*. Specifically, all segments with a length of 13 or 11 bp and excluding the terminal 3 base pairs were employed in our MD calculations for dsRNA or dsDNA, and the bending angle of a segment was determined by the angle between the tangents of the two end base steps along the central axis (22). In the calculations of bending angle probabilities *p*(*θ*), a bin width of 0.5 degree was employed for bending angle *θ*. Meanwhile, the bending energy can be also calculated from the MD trajectories by (41,63,64)


(3) \begin{eqnarray*}{\mathrm{\Delta }}{E}_{{\mathrm{bend}}}\left( \theta \right)/{k}_BT = - \ln \left( {p\left( \theta \right)/{\mathrm{sin}}\theta } \right).\end{eqnarray*}

### Calculating ion binding distributions on dsRNA and dsDNA

To understand the role of monovalent ions in the bending stiffness, we calculated the ion distributions around the dsRNA and dsDNA. In our calculations, the binding ions (including both cations and anions) per nucleotide were partitioned into those binding externally around phosphate groups (external binding ions), and those binding internally in major and minor grooves (internal binding ions), similar to (23,49). Specifically, the (internal) binding ions into the grooves were calculated as those within a helical distance of 8.5 Å and in the respective major/minor grooves, and the (external) binding ions around phosphate groups were calculated as those other than the ions in grooves and within a radial distance of 6 Å from phosphate atoms. Here, the helical distance of 8.5 Å was taken as the helical radii (∼10 Å) of dsRNA and dsDNA minus the radius (∼1.5 Å) of phosphate groups to better distinguish the binding ion distributions, and the radial distance of 6 Å from phosphate atoms corresponds to the second shell of Na^+^/Li^+^ distributions around phosphate atoms; see Supplementary Figure S9. Thus, the ion binding patterns were roughly classified into external binding ions (around phosphate groups) and internal binding ions (into major grooves or minor grooves).

Here, our calculated binding ions in the vicinity of dsRNA/dsDNA (23,29) somewhat differ from the binding ion atmosphere for dsRNA and dsDNA which describes the excess binding ions over bulk concentrations in whole solutions (17,56,65,66) and can be measured by the ion-counting experiments (67,68). Such calculations of binding ions were involved here since binding ions in the vicinity generally play a major role in the interactions with nucleic acids (29,49).

### Poisson–Boltzmann calculations for electrostatic contributions

To quantify the electrostatic contribution in bending elasticity of dsRNA and dsDNA, we calculated the electrostatic bending energy (Δ*E*_el_) of dsRNA and dsDNA using Adaptive Poisson–Boltzmann Solver (APBS) (69–71), a well-established Poisson-Boltzmann solver for biomolecules. In the calculations, the radii of ions (Na^+^ and Cl^−^) were set as 2 Å, and the dielectric constant of dsRNA and dsDNA was taken as 8, a value from the recent experiments and simulations (51,72), and that of solvent was taken as 78 (73). For the conformations from the MD simulations, all the atom charges including H atoms were involved in the electrostatic calculations according to the atom charge distributions from AMBER ff99bsc1+χOL3 force fields by PDB2PQR (69,74). Afterward, the electrostatic bending energy Δ*E*_el_ at bending angle *θ* is given by


(4) \begin{eqnarray*}\Delta {E}_{{\mathrm{el}}}\left( \theta \right) = {E}_{{\mathrm{el}}}\left( \theta \right) - {E}_{{\mathrm{el}}}\left( {\theta = 0} \right),\end{eqnarray*}

and the intrinsic (non-electrostatic) bending energy Δ*E*_nel_(*θ*) is given by


(5) \begin{eqnarray*}\Delta {E}_{{\mathrm{nel}}}\left( \theta \right) = \Delta {E}_{{\mathrm{bend}}}\left( \theta \right) - \Delta {E}_{{\mathrm{el}}}\left( \theta \right).\end{eqnarray*}

Here, Δ*E*_bend_(*θ*) can be obtained from the MD trajectories; see Eq. (3). According to the WLC model, the persistence length *P* can be calculated from bending energy (40,41,63,64)


(6) \begin{eqnarray*}{\mathrm{\Delta }}{E}_{{\mathrm{bend}}}\left( \theta \right)/{k}_BT = P{\theta }^2/2{L}_c.\end{eqnarray*}

Similarly, the electrostatic and intrinsic (non-electrostatic) persistence length *P*_el_ and *P*_nel_ can be calculated from the electrostatic and intrinsic (non-electrostatic) bending energies (Δ*E*_el_(*θ*) and Δ*E*_nel_(*θ*)) according to Eq. (6).

## Results and discussion

### High-precision MT measurements for *P*_RNA_ and *P*_DNA_ until very high monovalent salts

**Figure 2.**
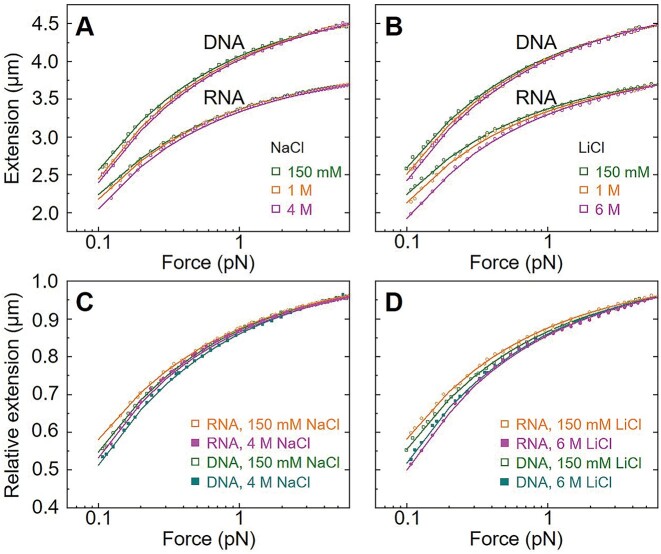
Effects of ion concentration on the force-extension curves of the dsDNA and dsRNA from the MT measurements. (**A**) Representative *F-x* curves of dsRNA and dsDNA at typical NaCl concentrations. (**B**) Representative *F-x* curves of dsRNA and dsDNA at typical LiCl concentrations. (**C**) Representative relative *F-x* curves of dsDNA and dsRNA at typical NaCl solutions. (**D**) Representative relative *F-x* curves of dsDNA and dsRNA at typical LiCl solutions. Each *F–x* curve is fitted to Eq. (1) (solid line), yielding bending persistence length *P* and contour length *L*_c_.

To quantify the relative bending stiffness of dsRNA and dsDNA, we characterized the bending persistence lengths, *P*_RNA_ and *P*_DNA_, of dsRNA and dsDNA at extensive monovalent salt concentrations by our high-precision MT experiments; see Figure 1A and Materials and methods. In particular, our measurements were extended to very high monovalent salt concentrations, such as 4 M NaCl and 6 M LiCl from near-physiological concentrations (150 mM NaCl and LiCl). At each salt condition, we repeated the measurements for at least ten molecules to obtain the averages and standard errors of *L_c_* and *P*. As shown in Figure 2, the WLC model works well for both dsRNA and dsDNA over the salt concentration ranges covered here.

**Figure 3.**
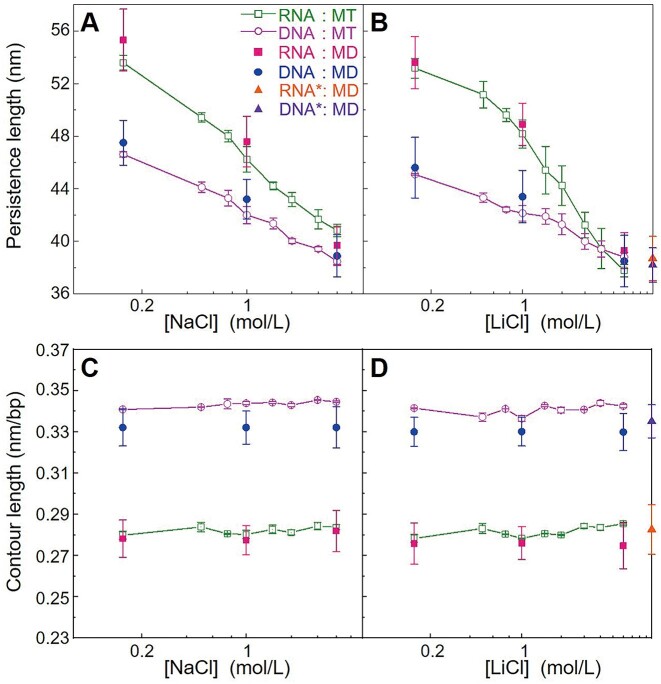
The persistence lengths of dsRNA and dsDNA are different at moderate salts but similar at very high salts with constant contour lengths. (**A**) The persistence length *P* as a function of NaCl concentrations. (**B**) The persistence length *P* as a function of LiCl concentrations. (**C**) The contour length *L*_c_ as a function of NaCl concentrations. (**D**) The contour length *L*_c_ as a function of LiCl concentrations. RNA* and DNA* denote the electrically ‘neutral’ dsRNA and dsDNA. The error bars denote the standard deviations around the mean values from the MT measurements using at least ten molecules or the values of four equal intervals in our MD trajectories.

The experimental *P*_RNA_ and *P*_DNA_ decreased with the increase of NaCl and LiCl concentrations ([NaCl] and [LiCl]); see Figures 3A and B and Supplementary Table S1. Meanwhile, as shown in Figure 3 and Supplementary Table S1, the decrease of *P*_RNA_ with the increase of salt concentrations is more rapid than that of *P*_DNA_. Specifically, when [NaCl] was increased from 150 mM to 4 M, *P*_RNA_ decreased from ∼54 nm to ∼41 nm, and *P*_DNA_ decreased from ∼47 to ∼38 nm. Similarly, with the increase of [LiCl] from 150 mM to 6 M, *P*_RNA_ decreased from ∼53 to ∼38 nm, and *P*_DNA_ decreased from ∼45 to ∼39 nm. Moreover, *P*_RNA_ and *P*_DNA_ at 150 mM NaCl were similar with those at 150 mM LiCl, and at near-physiological monovalent salts (e.g. ∼150 mM), *P*_RNA_ was visibly larger than *P*_DNA_, in agreement with previous single-molecule experiments (10,27–29). It is interesting that when monovalent salt became very high (>∼4 M), *P*_RNA_ and *P*_DNA_ converged to ∼39 nm (e.g. at 4 M NaCl and 6 M LiCl), given that *P*_RNA_ was visibly larger than *P*_DNA_ at near-physiological salt. This phenomenon resulted from the more rapid decrease of *P*_RNA_ with salt concentration than that of dsDNA; see Figures 3A and B and Supplementary Table S1. Additionally, as shown in Figures 2 and 3, the experimental *L_c_* almost kept constant at ∼0.34 nm/bp for dsDNA and ∼0.28 nm/bp for dsRNA across the investigated salt concentration ranges, regardless of the ion types. It should be noted that a slight difference remains at 4 M NaCl, but a crossover occurs at 4 M LiCl, and at 6 M LiCl, the order of *P*_RNA_ and *P*_DNA_ is reversed.

It would be interesting to examine the well-known Odijk–Skolnick–Fixman (OSF) and Barrat-Joanny (BJ) models which describe the monovalent-salt dependence of persistence length of a polyelectrolyte (75–77), through a comparison with our MT measurements up to very high salt concentration. As shown in Supplementary Figure S6, we found the BJ model works slightly better than the OSF model for our MT data, i.e. the mono-salt dependence of our measured *P*_RNA_/*P*_DNA_ is somewhat stronger than that described by the OSF model, consistent with previous works (22,27). Interestingly, it is noted that the non-electrostatic persistence length *P*_nel_’s fitted from the BJ model for dsDNA (∼38.5 nm) and dsRNA (∼38.4 nm) are very close to the *P* values (∼39 nm) from our MT experiments at very high salts. Please see Supplementary Figure S6 and Supplementary Table S2 for the details.

### Bending persistence lengths of dsRNA and dsDNA from MD simulations

**Figure 4.**
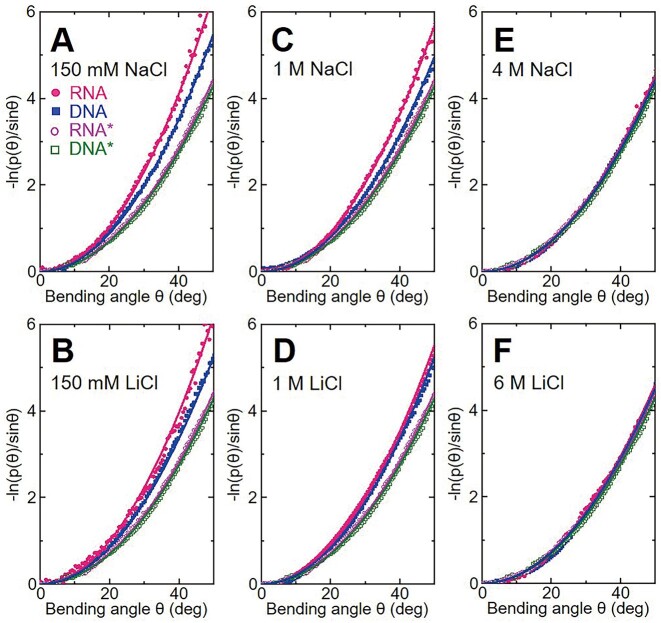
The bending angle distributions of dsDNA and dsRNA are different at moderate salts but similar at very high salts. The bending angle distributions *p*(*θ*) versus bending angle *θ* over a given length *L*_c_ of the dsRNA and dsDNA. RNA* and DNA* denote the electrically ‘neutral’ dsRNA and dsDNA. Here, for convenience, the segments with 13-bp dsRNA and 11-bp dsDNA, which have a similar contour length of ∼3.3 nm, were used in our calculations for dsRNA and dsDNA, respectively. The bending persistence length *P* of dsRNA and dsDNA can be calculated by fitting *p*(*θ*) according to Eq. (2).

To explore the origin of why dsRNA has visibly higher bending stiffness than dsDNA at near-physiological salts and the mechanism for the bending stiffness similarity of dsRNA and dsDNA at very high monovalent salts, we performed the all-atom MD simulations for the dsRNA and dsDNA at typical salt concentrations as well as for the electrically ‘neutral’ dsRNA* and dsDNA*. In the MD simulations, we selected six typical salt concentrations including 150 mM NaCl, 150 mM LiCl, 1 M NaCl, 1 M LiCl, 4 M NaCl, and 6 M LiCl for both dsRNA and dsDNA. Based on the bending angle-dependent probability *p*(*θ*) from the MD trajectories, *P*_RNA_ and *P*_DNA_ at different salt concentrations were calculated by fitting *p*(*θ*) according to Eq. (2), as shown in Figure 4 and Supplementary Figure S10. Please see Materials and methods for the details of the MD simulations and the calculations of *P*_RNA_ and *P*_DNA_.

As shown in Figure 3, the calculated *P*_RNA_’s and *P*_DNA_’s from the MD simulations were in good agreement with the MT measurements and converged at very high salt concentrations. Specifically, for NaCl solutions, the calculated *P*_RNA_’s were ∼55 nm at 150 mM NaCl and ∼40 nm at 4 M NaCl, and the calculated *P*_DNA_’s were ∼48 nm at 150 mM NaCl and ∼39 nm at 4 M NaCl. For LiCl solutions, the calculated *P*_RNA_’s were ∼54 nm at 150 mM LiCl and ∼39 nm at 6 M LiCl, and the *P*_DNA_’s were ∼46 nm at 150 mM LiCl and ∼39 nm at 6 M LiCl. Namely, our MD simulations also reproduce the major features of salt-dependent *P*_RNA_ and *P*_DNA_: dsRNA has visibly larger bending stiffness than dsDNA at near-physiological salt concentrations, and *P*_RNA_ and *P*_DNA_ converge at very high monovalent salts. The contour lengths derived from our MD simulations are almost independent on salt concentration in analogy with our MT measurements, although the contour lengths from the MD simulations are very slightly smaller than the experimental values.

Furthermore, to explore the intrinsic bending stiffness of dsRNA and dsDNA, we performed the all-atom MD simulations for electrically ‘neutral’ dsRNA* and dsDNA*; see Materials and methods for details of building the dsRNA*and dsDNA*. As shown in Figure 3 and Supplementary Table S1, it is very interesting that the calculated *P*_RNA_ and *P*_DNA_ were both close to ∼39 nm, the value close to that from the MT experiments and MD simulations for normal dsRNA/dsDNA at 6 M LiCl and 4 M NaCl. This suggests that the bending stiffness of dsRNA and dsDNA at very high monovalent salts approximately converged to the same intrinsic (non-electrostatic) one. Therefore, the different bending stiffness of dsRNA and dsDNA at near-physiological monovalent salt concentrations should mainly result from the different electrostatic interactions which can be fully compensated by very high monovalent salts. It is also interesting to examine the structures of dsRNA* and dsDNA* by calculating seven major helical parameters. We found that the seven major helical parameters of dsRNA* and dsDNA* are similar to those of dsRNA and dsDNA at different salts, respectively; see Supplementary Figure S11. This suggests that dsRNA* and dsDNA* kept A-form and B-form respectively, in analogy to dsRNA and dsDNA at different monovalent salts. Moreover, dsRNA* and dsDNA* are indeed electrically neutral, as illustrated in Supplementary Figure S11H.

### Structure and salt-concentration dependent ion-binding distributions from MD simulations

To deeply understand the above-described salt-dependent bending stiffness of dsRNA and dsDNA, we calculated the ion binding distributions for dsRNA and dsDNA and the relationship between bending angle and binding ions.

**Figure 5.**
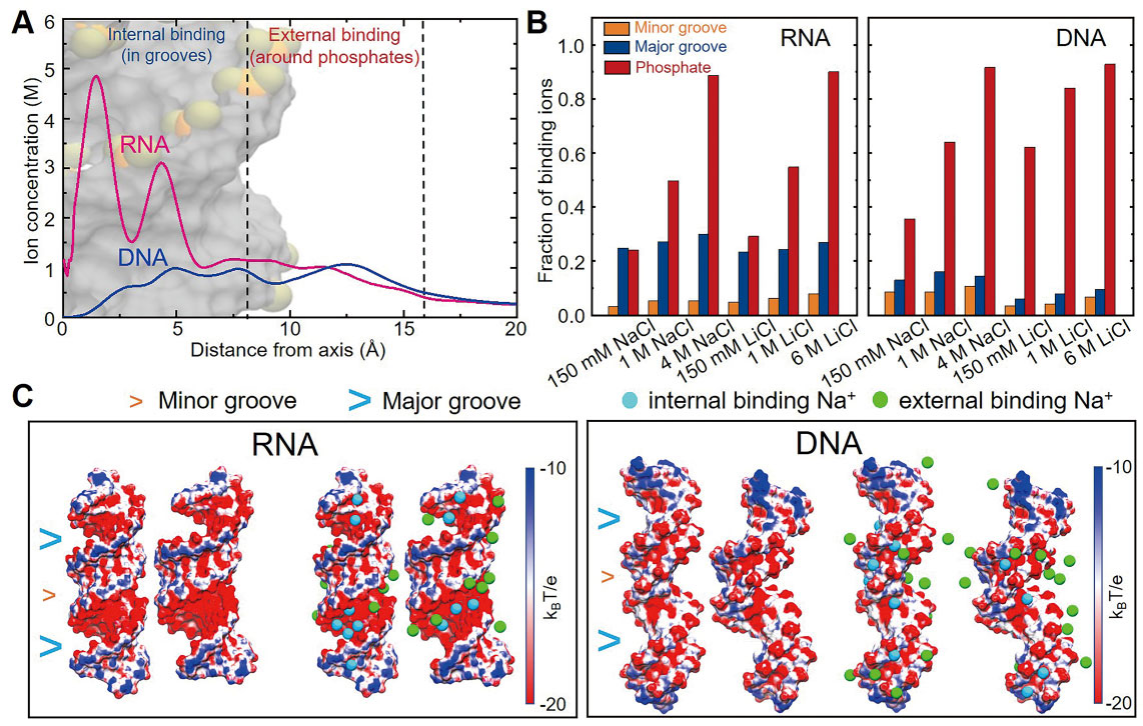
Fractions of binding ions around phosphates for dsRNA and dsDNA are different at moderate salts but similar at very high salts. (**A**) The radial concentration distributions of Na^+^ around the dsRNA and dsDNA obtained from our MD simulations (17,49). At a large radial distance, the Na^+^ concentrations converge to the desired concentration of 150 mM. (**B**) The charge fractions of binding cations (over anions) per nucleotide from our MD simulations at different ion conditions for dsRNA (left) and dsDNA (right). Please see Supplementary Table S4 for the details. (**C**) Representative straight and bent structures and their ion distributions of dsRNA (left) and dsDNA (right) showing the surface electrostatic potentials at 150 mM NaCl by the PB solver of APBS (70).

**Figure 6.**
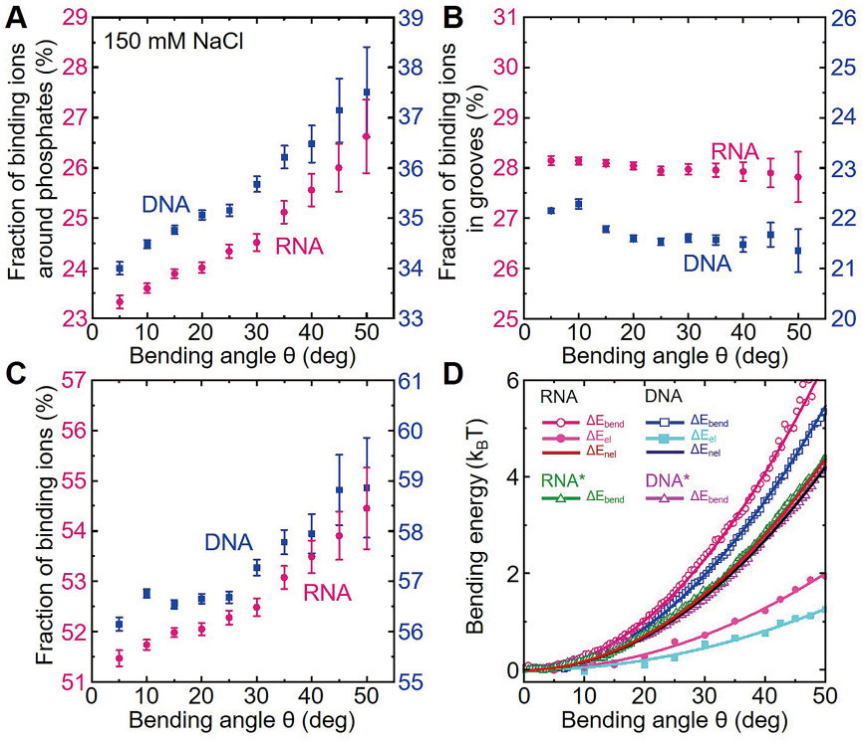
Ion binding around phosphates plays a major role in dsRNA and dsDNA bending. (**A**) Average charge fractions of (externally) binding ions around phosphates for dsRNA (red) and dsDNA (blue) as functions of bending angle. (**B**) Average charge fractions of (internally) binding ions in grooves for dsRNA (red) and dsDNA (blue) as functions of bending angle. (**C**) Average charge fractions of total binding ions for dsRNA (red) and dsDNA (blue) as functions of bending angle. (**D**) The bending energy Δ*E*_bend_, electrostatic bending energy Δ*E*_el_, and non-electrostatic bending energy Δ*E*_nel_ versus bending angle *θ* of dsRNA and dsDNA. Δ*E*_bend_ was calculated by Eq. (3); Δ*E*_el_ was calculated by the APBS for the MD conformations (70); Δ*E*_nel_ was calculated by Eq. (5) with the shown fitted lines for Δ*E*_bend_ and Δ*E*_el_.

We found that *P*_RNA_ and *P*_DNA_ are closely coupled to the ion binding patterns around dsRNA and dsDNA, by comparing Figures 3 and 5; see also Supplementary Table S4. Specifically, at 150 mM monovalent salts, cations bind comparably in the major groove and around phosphate groups for A-form dsRNA, while preferred to bind around phosphate groups rather than in grooves for B-form dsDNA. This is attributed to the much stronger electrostatic potential in the major groove for dsRNA than for dsDNA; see Figure 5C for the surface electrostatic potentials from APBS and ion distributions for straight/bent dsRNA and dsDNA from our MD simulations (17). This naturally results in visibly lower ion neutralization effects for phosphate groups of dsRNA than for those of dsDNA, e.g. charge fractions of binding Na^+^ over Cl^−^ around phosphate groups is ∼0.24 for dsRNA and is ∼0.36 for dsDNA at 150 mM NaCl. This causes the stronger electrostatic bending repulsion for dsRNA and the higher *P*_RNA_ than *P*_DNA_ at near-physiological salt concentrations since the negative charges of dsRNA/dsDNA mainly exist in phosphate groups. Moreover, compared with Na^+^, Li^+^ binds slightly more strongly around the phosphate groups for both dsRNA and dsDNA due to the stronger binding affinity of Li^+^ with a smaller ionic size than Na^+^; see also Supplementary Figure S9 (78). When [NaCl]/[LiCl] is increased to very high concentrations (e.g. 4M NaCl), the ion binding was greatly enhanced to a very high degree for both dsRNA and dsDNA, and the enhancement in ion binding around phosphate groups for dsRNA is more pronounced than that for dsDNA. Namely, the increase in charge fraction of binding Na^+^ (over Cl^−^) around phosphate groups from ∼0.24 to ∼0.89 for dsRNA is more pronounced than that from ∼0.36 to ∼0.91 for dsDNA, and the concentration-dependent trend of ion binding for LiCl is similar to that for NaCl; see Figure 5B. This is attributed to the limited binding space of the narrow and deep major groove of dsRNA for cations and associated anions at very high concentrations (17), and the excess binding ions would bind around negatively charged phosphate groups for dsRNA. With the increase of ion concentration, internal binding ions increase slightly for both dsRNA and dsDNA, and such increase is slightly more pronounced for dsRNA than for dsDNA due to the stronger electrostatic potential in the major groove of dsRNA; see Figure 5B. Very important, it is noted that the dsRNA and dsDNA are both around full-neutralized for phosphate groups (charge fraction of binding ions ∼0.91) and the whole molecules (charge fraction of binding ions ∼1.19). Furthermore, we examined the relationships between bending angle and binding ion distributions (internal, external, and total binding ions) for both dsRNA and dsDNA. As shown in Figure 6A–C and Supplementary Figure S12A–F, the dependence of bending on external binding ions (around phosphate groups) makes the dominant contribution to that of bending on total binding ions, rather than that on internal binding ions, for both dsRNA and dsDNA. Less/more external binding ions (around phosphate groups) correspond to the dsRNA and dsDNA structures with small/large bending angles, respectively; see Figures 5C and 6A–C. The major role of external binding ions rather than internal ones is also reflected in the relationships between (external/internal/total) binding ions and the fluctuation of total groove width, as shown in Supplementary Figure S19.

Thus, the larger *P*_RNA_ than *P*_DNA_ at near-physiological monovalent salt is mainly attributed to the weaker external ion binding around phosphate groups, and the similar intrinsic *P*_RNA_ and *P*_DNA_ at very high salt concentrations are due to the approximate full-neutralization of external ion binding around phosphate groups and the whole molecules.

### Electrostatic and intrinsic bending stiffness of dsRNA and dsDNA from PB calculations

To quantify the electrostatic and intrinsic (non-electrostatic) contributions to the bending stiffness of dsRNA and dsDNA at physiological monovalent concentrations (e.g. 150 mM NaCl), we calculated the electrostatic bending energy Δ*E*_el_ with the APBS (70) and the intrinsic bending energy Δ*E*_nel_ based on total bending energy Δ*E*_bend_ from the MD simulations through Eqs. (3–6); see Materials and methods for the details. As shown in Figure 6D, the following features can be found. First, Δ*E*_bend_ increases with the increase of bending angle *θ* for both dsRNA and dsDNA in a quadratic way, consistent with the WLC model (41,63). Second, Δ*E*_bend_ increases more strongly for dsRNA than for dsDNA, in consistency with the above-shown higher *P*_RNA_ than *P*_DNA_ at 150 mM NaCl; see Figures 3 and 4 and Supplementary Table S5. Third, this phenomenon is attributed to the weaker ion binding to phosphate groups as discussed above and consequently stronger electrostatic bending repulsions for A-form dsRNA than for B-form dsDNA. Fourth, the calculated electrostatic persistence lengths *P*_el_’s of dsRNA and dsDNA from the PB calculations by fitting Δ*E*_el_(*θ*) to Eq. (6) are ∼17 and ∼10 nm, respectively. Fifth, as shown in Figure 6D, the intrinsic bending energies Δ*E*_nel_ of dsRNA and dsDNA are nearly identical over the wide range of bending angle *θ*, and the corresponding intrinsic persistence lengths *P*_nel_’s of dsRNA and dsDNA converged to the similar value (∼39 nm for dsRNA and ∼38 nm for dsDNA) corresponding with those from the MT experiments and the MD simulations for dsRNA and dsDNA at very high monovalent salts and for the electrically ‘neutral’ dsRNA* and dsDNA*. This suggests the difference between *P*_DNA_ and *P*_RNA_ at near-physiological salt concentration comes from the different electrostatic contributions between dsRNA and dsDNA.

Therefore, our electrostatic calculations confirmed that the higher *P*_RNA_ than *P*_DNA_ at near-physiological monovalent salts is mainly attributed to the electrostatic interactions, and *P*_RNA_ and *P*_DNA_ converged to the same intrinsic one at very high monovalent salts. It is noted that the PB calculations were stable against the slight change around the used dielectric constant of 8 for dsRNA/dsDNA from experiments and simulations (51,72) and against the number of conformations from the MD simulations; please see Supplementary Figures S13, S14, and Supplementary Table S6 for the details.

### Why do A-form dsRNA and B-form dsDNA have similar intrinsic bending stiffness?

**Figure 7.**
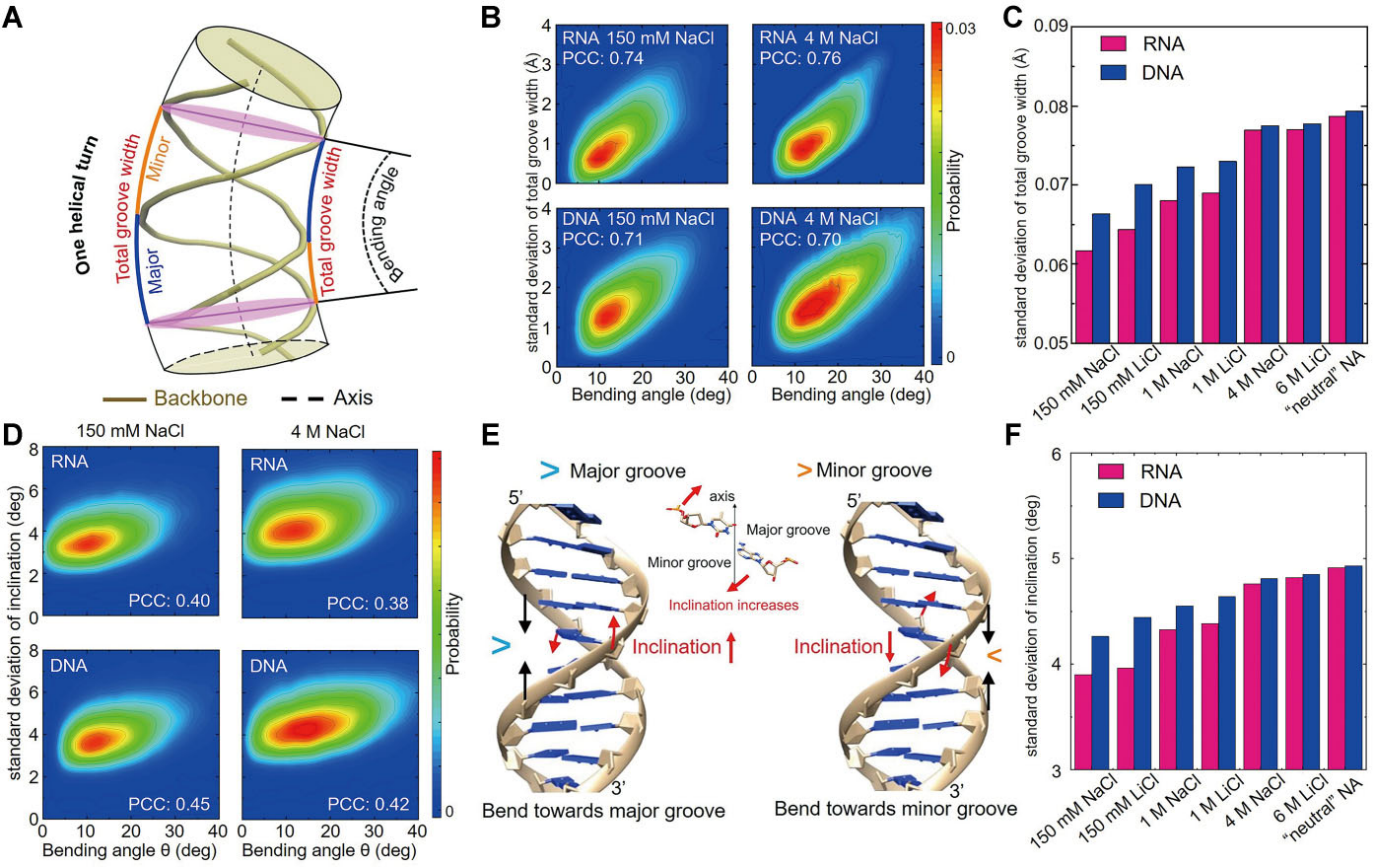
Different fluctuations of the total groove widths around axis and base-pair inclinations are the origin of the different bending stiffness of dsDNA and dsRNA at physiological monovalent salts. (**A**) An illustration of the axial grooves of a dsRNA or dsDNA. (**B**) The relationships between the standard deviation of the total groove width and bending angle *θ* at 150 mM and 4 M NaCl for dsRNA and dsDNA, where PCCs stand for the Pearson correlation coefficients; please see Supplementary Figure S17 for those at other salts. (**C**) The standard deviation of the axial total groove width per unit contour length at different ionic conditions, and the ‘neutral’ NA stands for the electrically ‘neutral’ dsRNA* and dsDNA*. (**D**) The relationships between the standard deviation of inclination and bending angle *θ* at 150 mM and 4 M NaCl for dsRNA and dsDNA, where PCCs stand for the Pearson correlation coefficients; please see Supplementary Figure S18 for the PCCs at other salts. (**E**) An illustration for that bending towards major groove and towards minor groove would lead to an increase and a decrease in inclination, respectively; see Supplementary Figure S20 for the relationships between base-pair inclination and bending angle for bending towards major grooves or minor grooves (79). (**F**) The standard deviation of inclination at different ionic conditions, and the ‘neutral’ NA stands for the electrically ‘neutral’ dsRNA* and dsDNA*.

Why do A-form dsRNA and B-form dsDNA with different helical structures have a similar intrinsic bending stiffness at very high monovalent salts or full-neutralization conditions? This interesting question still requires to be addressed. Since helix bending generally involves the change of major groove width *D* or minor groove width *d*, we examined the correlation between bending angle *θ* and *D* and that between bending angle *θ* and *d* for dsRNA and dsDNA, respectively. For convenience, we used the axial widths of the major groove and minor groove since such axial widths seem to be directly related to the bending of dsRNA and dsDNA, as shown in Figure 7A; also see Supplementary material for the calculations of axial groove widths. Here, axial groove width means the groove width along the axial direction that spans major/minor grooves and thus there are axial major groove width, axial minor groove width, and axial total groove width, respectively, as shown in Figure 7A. In the following, we would use major groove width, minor groove width, and total groove width to stand for axial major groove width, axial minor groove width, and axial total groove width, respectively.

First, we found that for dsRNA and dsDNA, the bending angle *θ* correlates very weakly with the fluctuation of *d* while correlated modestly with the fluctuation of *D*, and such correlation is slightly stronger for dsRNA; see Figure 7B and Supplementary Figures S15-S17. Second, it is very interesting that the bending angle *θ* correlated strongly and positively to the fluctuation of the total width of the major groove and minor groove (namely, total groove width *D*+ *d*) for both dsRNA and dsDNA; see Figures 7A and B and Supplementary Figures S15–S17. Thus, we obtained the interesting conclusion that the major groove and minor groove together determine the bending stiffness for both dsRNA and dsDNA beyond the major groove or the minor groove separately. Moreover, as shown in Figure 7C, the fluctuation of total groove width *D*+ *d* of dsRNA is smaller than that of dsDNA at 150 mM monovalent salt, corresponding to the higher bending stiffness of dsRNA than that of dsDNA (i.e. larger *P*_RNA_ than *P*_DNA_). When monovalent salt becomes very high or dsRNA/dsDNA becomes electrically ‘neutral’, the fluctuations of *D*+ *d* of dsRNA and dsDNA converged together, corresponding to the similar *P*_RNA_ and *P*_DNA_ compared with those at 150 mM monovalent salts. Therefore, the similar bending stiffness of dsRNA and dsDNA at very high monovalent salts is attributed to the similar fluctuation of total groove width, although dsRNA and dsDNA have very different A-form and B-form helical structures.

Furthermore, we would examine the relationships between bending and more microscopic structure parameters, by calculating Pearson Correlation Coefficients (PCCs) between bending angle and 16 important base-pair parameters as well as their fluctuations along a bent conformation. As shown in Figure 7D and Supplementary Figure S18 and Supplementary Table S7, the correlations between bending angle and the microscopic structural parameters and their fluctuations along a bent conformation are generally rather weak, while those between bending angle and the fluctuation of base-pair inclination along a bent conformation are relatively strong (PCC ∼0.4) for both dsRNA and dsDNA. This suggests that the fluctuation of base-pair inclination along a bent conformation correlates apparently with dsRNA and dsDNA bending. This is because a helix bending generally involves bending's towards major groove and minor groove (79) while bending's around a base pair towards major groove and towards minor groove generally correlate to the increase and decrease of the base-pair inclination; see Figure 7E and Supplementary Figure S20 for the details. Therefore, the different inclinations corresponding to bending's towards different directions make the major contribution to a helix bending, and a larger bending angle is correlated to more different inclinations for a helix and stronger fluctuation of inclination along the helix as discussed above.

Correspondingly, as shown in Figure 7F, the fluctuation of inclination for dsRNA is smaller than that for dsDNA at 150 mM monovalent salts, corresponding to the larger *P*_RNA_ than *P*_DNA_. When monovalent salt becomes very high or dsRNA/dsDNA becomes electrically ‘neutral’, the fluctuation of inclination for dsRNA and dsDNA converge, corresponding to the decreased similar *P*_RNA_ and *P*_DNA_, compared with those at 150 mM monovalent salts. Additionally, the correlations between total groove widths and the fluctuation of inclinations were shown to be relatively strong for both dsRNA and dsDNA; see Supplementary Figure S19E–H.

### Origin of different bending stiffness between dsRNA and dsDNA

Based on the above high-precision MT measurements, all-atom MD simulations, and PB calculations, we could obtain an overall understanding of the salt-dependence of the relative bending stiffness of dsRNA and dsDNA based on the ion binding distributions and the fluctuation of total groove widths and base-pair inclinations for dsRNA and dsDNA. At near-physiological monovalent salts, compared with dsDNA, ions rarely bind around the phosphate groups of dsRNA due to the deep/narrow major groove, causing weaker ionic neutralization for phosphate groups and stronger electrostatic repulsions between phosphate groups for dsRNA. This leads to weaker fluctuation of total groove width and base-pair inclination, and consequently, the higher bending stiffness of dsRNA than dsDNA. As salt concentration is increased, the major groove of dsRNA would be fulfilled by cations and associated anions, and the excess ions would become binding around the phosphate groups of dsRNA. When salt concentration becomes high enough, dsRNA and dsDNA are around full-neutralized for phosphate groups and the whole molecules, and the fluctuations of total groove widths and base-pair inclination of dsRNA and dsDNA become nearly identical despite their different helical structures. This results in a nearly identical (intrinsic) *P*_RNA_ and *P*_DNA_ for both dsRNA and dsDNA at very high salts.

## Conclusion

In summary, we combined high-precision MT experiments, all-atom MD simulations, and PB calculations to quantify the bending persistence lengths of dsRNA and dsDNA at varying monovalent salt concentrations and to explore the origin of the different bending stiffness of A-form dsRNA and B-form dsDNA at physiological monovalent salt concentration. First, our MT experiments and all-atom MD simulations revealed that while dsRNA appears visibly stiffer in bending than dsDNA at near-physiological monovalent salts, dsRNA and dsDNA have a similar bending persistence length of ∼39 nm at very high monovalent salts. Second, our all-atom MD simulations and PB calculations showed that the higher bending stiffness of dsRNA than dsDNA at physiological monovalent salts is attributed to the apparently weaker ion binding around phosphate groups, and dsRNA and dsDNA can be around fully-neutralized by very high monovalent salts, causing the similar intrinsic bending stiffness at very high salts. Third, we found that despite the different helical structures, the similar intrinsic bending stiffness of A-form dsRNA and B-form dsDNA (e.g. at very high salt concentrations) is attributed to the similar fluctuations of the total groove width and the similar fluctuation of base-pair inclination for dsRNA and dsDNA. Our results would be very helpful for understanding the biological functions of RNA and DNA and for building RNA and DNA nanostructures, since the bending stiffness of dsRNA and dsDNA is leadingly coupled to gene packing, complexing with proteins, and assembly into nanostructures.

## Supplementary Material

gkae063_Supplemental_File

## Data Availability

The data underlying this article are available in the article and its online Supplementary material.
