# Supplementary material for: The origin of different bending stiffness between double-stranded RNA and DNA revealed by magnetic tweezers and simulations
Source: Nucleic Acids Res. 2024 Feb 7;52(5):2519–29. doi: 10.1093/nar/gkae063 (PMC10954459; doi:10.1093/nar/gkae063)
Supplement: gkae063_Supplemental_File [file gkae063_supplemental_file.docx]

**Supporting Information for**

**The origin of different bending stiffness between double-stranded RNA and DNA revealed by magnetic tweezers and simulations**

Hai-Long Dong^1, #^, Chen Zhang^1, #^, Liang Dai^2^, Yan Zhang^3,^ *, Xing-Hua Zhang^1,^ *, and Zhi-Jie Tan^1,^ *

^1^School of Physics and Technology, College of Life Sciences, Renmin Hospital of Wuhan University, Wuhan University, Wuhan 430072, China

^2^Department of Physics, City University of Hong Kong, Hong Kong 999077, China

^3^Department of Clinical Laboratory, Renmin Hospital of Wuhan University, Wuhan 430072, China

^#^ Hai-Long Dong and Chen Zhang contributed equally to this work.

* To whom correspondence should be addressed.

zjtan@whu.edu.cn, [zhxh@whu.edu.cn](mailto:zhxh@whu.edu.cn) or peneyyan@whu.edu.cn

**Measurements of the force-extension curves of RNA and DNA by magnetic tweezers**

We conducted all MT measurements in an ultra-clean room to prevent RNA degradation and maintained a constant temperature of 22 ^o^C to minimize draft which was mainly caused by temperature fluctuations during the day-long experiments. We labeled each DNA or RNA molecule with a biotin and a digoxigenin group at either end. Details of the preparation of the RNA and DNA constructs were similar to our previous work (1) but with a different 13,751-bp sequence (Figure S1). After attaching polystyrene beads (3 μm diameter) to the glass surface as reference points, we anchored the digoxigenin-labeled ends of the RNA or DNA molecules to the anti-digoxigenin-coated glass surface. Subsequently, we passivated the glass surfaces overnight using 2% bovine serum albumin (Sigma-Aldrich) in phosphate-buffered saline (PBS). We then attached superparamagnetic microbeads (Dynabeads MyOne Streptavidin C1) to the biotin-labeled ends of the DNA or RNA molecules in PBS. We conducted all MT measurements using buffers containing 1 mM Tris-HCl pH 7.5 supplemented with various concentrations of NaCl or LiCl.

To acquire accurate force-extension curves for DNA and RNA across a broad range of salt concentrations, we implemented several specific strategies in our MT experiments. (1) Correction based on the buffer's refractive index. Since the buffer's refractive index (n_b_) influenced the diffraction pattern of the paramagnetic beads, we measured n_b_ for each salt concentration using a refractometer (PAL-RI, ATAGO Japan). We then recalibrated the bead's diffraction pattern at each salt concentration using a piezo objective scanner and recalculated the extension for each DNA or RNA molecule based on the measured n_b_ (Figure S2). (2) Selection of the paramagnetic beads. We selected to use DNA or RNA molecules anchored to the center bottom of the beads to minimize errors in the measured extension resulting from discrepancies between the axis of the force applied to the bead and the axis of the DNA/RNA molecule (Figure S3). (3) Determining the glass top's height. To obtain the absolute extension (i.e., the height difference between the bottom of the paramagnetic bead and the top of the functionalized glass surface) of the DNA and RNA, we determined the glass top's height at zero force, where the paramagnetic bead touched the glass (Figure S4). (4) Determining the applied force. To determine the exact applied force (*F*) at each magnets' height (*d*), we fitted *F* as a function of *d* for each paramagnetic bead (Figure S5). (5) To prevent the interactions between the DNA, the glass, and the beads which may lead to shorter contour lengths, we passivated all surfaces using mPEG-Succinimidyl Valerate ester (MW 2K, Hunan Huateng Pharmaceutical) in 100 mM sodium bicarbonate solution for two hours before the measurement of force-extension curves (2).

**All-atom molecular dynamics (MD) simulations for electrically “neutral” dsRNA and dsDNA**

To model the bending elasticity of dsRNA and dsDNA without electrostatic interactions, we built the electrically “neutral” dsRNA and dsDNA by adding a charge of +1e to each phosphate group, i.e., the charges of phosphate groups were artificially reduced in the same proportions to equivalently add a +1e charge to each phosphate group, and the charges of other atoms were kept unchanged (3,4). Specifically, in our MD simulations, we reduced the atom charges of phosphate groups including four oxygen (OP1, OP2, O3’, O5’) atoms and one phosphorous (P) atom in the same proportions (29.04% for dsRNA and 28.82% for dsDNA) while the partial charges of other atoms were unchanged to avoid the change of base pair stacking/pairing of dsRNA and dsDNA; see the detailed partial charges of the atoms of phosphate groups in Table S3. The MD simulations for the “neutral” dsRNA and dsDNA were performed until 600 ns at 150 mM NaCl according to the procedure described in the main text. For convenience, we labeled the electrically “neutral” dsRNA and dsDNA as dsRNA* and dsDNA*, respectively.

**Helical parameters of dsRNA and dsDNA**

To avoid the end effect of the short duplexes, three base pairs at each end of dsRNA and dsDNA were removed in our analyses of the MD trajectories (5-9). In our analyses, the helical and geometrical parameters of dsRNA and dsDNA were obtained with the use of Curves+ (10,11).

**Poisson-Boltzmann calculations for electrostatic contribution**

To quantify the electrostatic contribution in bending elasticities of dsRNA and dsDNA, we calculated the electrostatic bending energy (Δ*E*_el_) of dsRNA or dsDNA using APBS (12-14), a well-established Poisson-Boltzmann solver for biomolecules. In the calculations, the radii of ions (Na^+^ and Cl^−^) were set as 2 Å, and the dielectric constant of dsRNA and dsDNA was taken as 8, a value from the recent experiments and simulations, and that of solvent was taken as 78 (15,16). For the conformations from the MD simulations, all the atom charges including H atoms were involved in the electrostatic calculations according to the atom charge distributions from AMBER ff99bsc1+χ_OL3_ force fields by PDB2PQR (17). Afterward, the electrostatic bending energy ∆*E*_el_(*θ*) at bending angle *θ* over *L*_c_ is given by

$\Delta E_{\mathrm{el}}(\theta)=E_{\mathrm{el}}(\theta)-E_{\mathrm{el}}(\theta=0)$. (S1)

Here, *E*_el_(*θ*) was calculated from APBS. Moreover, the intrinsic (non-electrostatic) bending energy ∆*E*_nel_(*θ*) is given by

$\Delta E_{\mathrm{nel}}(\theta)=\Delta E_{\mathrm{bend}}(\theta)-\Delta E_{\mathrm{el}}(\theta)$. (S2)

Here, ∆*E*_bend_(*θ*) can be obtained from the MD trajectories. According to the WLC model, the persistence length *P* can be calculated from the bending energy

${\Delta E_{\mathrm{bend}}\left( \theta\right)}/{k_{B}T}=P\theta^{2}/2L_{c}$. (S3)

Thus, the electrostatic and intrinsic (non-electrostatic) persistence length *P*_el_ and *P*_nel_ can be calculated from the electrostatic and intrinsic (non-electrostatic) bending energies; see Eqs. (S1) and (S2). In our calculations, the bending angle interval is 5° from 0° to 40° and for more accuracy at large bending the interval of 2.5° from 42.5° to 50° is used and then the box bin is set as 1°. Meanwhile, the conformations in each box are randomly selected. The total number of conformations used in the electrostatic bending energy calculations for dsRNA and dsDNA at different bending angles are listed in Table S6. Meanwhile, as shown in Figure S14, Δ*E*_el_(*θ*)’s for different bending angles converge gradually against the number of enough conformations taken from the MD simulations.

**Calculating axial groove width of dsRNA and dsDNA**

Given the very different helical structures, how do dsRNA and dsDNA have similar bending rigidities at high monovalent salt/electrically neutral conditions? We made the analyses based on the MD simulations to answer the interesting question. As shown in Figure 7A in the MS, the axial groove widths are introduced and such groove width is the summation of the major groove width and minor groove width in the direction of the helical axis. The axial major groove width can be calculated as the distance between the backbone phosphate atoms on two adjacent strands crossing the major groove along the helical axis, and similarly, the axial minor groove width can be calculated as that crossing the minor groove along the helical axis. Afterward, the axial groove width is the summation of the axial major groove width and axial minor groove width. In practice, for more accurate calculations of axial major and minor groove widths, we used the three-dimensional linear interpolations to produce more continuously distributed atoms along backbones based on the coordinates of phosphate atoms.

**Calculating the relationships between bending angle and base-pair parameters of dsRNA and dsDNA**

To avoid the end effect of the short duplexes, three base pairs at each end of dsRNA and dsDNA were removed in our analyses of the MD trajectories. In our analyses, the helical and geometrical parameters of dsRNA and dsDNA were obtained with the use of Curves+. Specially, for one structure from MD simulations, we can obtained an average bending angle for different segments with counter length of ~3.3 nm, an average base-pair parameter and its standard deviation. The base-pair parameters contain 6 local base-pair parameters (shear, buckle, stretch, propeller, stagger, and opening), 6 local base-pair step parameters (shift, tilt, slide, roll, rise, and twist), 4 helical base-pair axis parameters (x-displacement, y-displacement, inclination, and tip). It should be noted that the average value and its standard deviation for a base-pair parameter were obtained for one conformation. The relationships between bending angle and base-pair parameters or their standard deviations were obtained; see Figures 7D in the MS and Figure S18 and Table S7 in the SI.


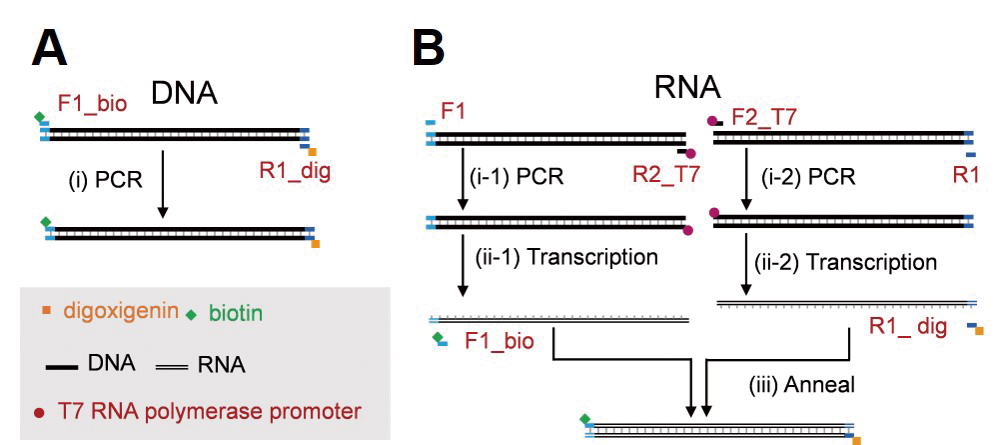


**Figure S1.** Preparation of DNA and RNA constructs for MT experiments. The DNA and RNA had the same sequences (57.2% GC, 6350-20100 bp of lambda DNA). The name of primers used in each step were indicated in red text in the figure.

(A) The DNA construct.

(i) We amplified the DNA using lambda DNA as the template and F1-bio and R1-dig as the primers.

(B) The RNA construct.

(i-1) We amplified the DNA using lambda DNA as the template and F1 and R2-T7 as the primers. (ii-1) hen, we transcribed the PCR product using T7 RNA polymerase.

(i-2) We amplified the DNA using lambda DNA as the template and F2_T7 and R1 as the primers. (ii-2) Then, we transcribed the PCR product using T7 RNA polymerase.

(iii) We annealed the four single-stranded comments together. These included the two long ssRNAs generated from transcription and the two primers F1_bio and R1_dig.

We synthesized all the primers with the following sequences.

F1-bio: Bio-ATTTACGCCGGGATATGTCAAGC

R1-dig: Dig-AGTCAGTTGCATCAGTCACAAGGG

F1: ATTTACGCCGGGATATGTCAAGC

R2-T7: TAATACGACTCACTATAGGGGCGACCTGAGCAGCTGATGCACTG

F2_T7: TAATACGACTCACTATAGGGAAGCATGAAGTGAATCCGCAGATGAC

R1: AGTCAGTTGCATCAGTCACAAGGG


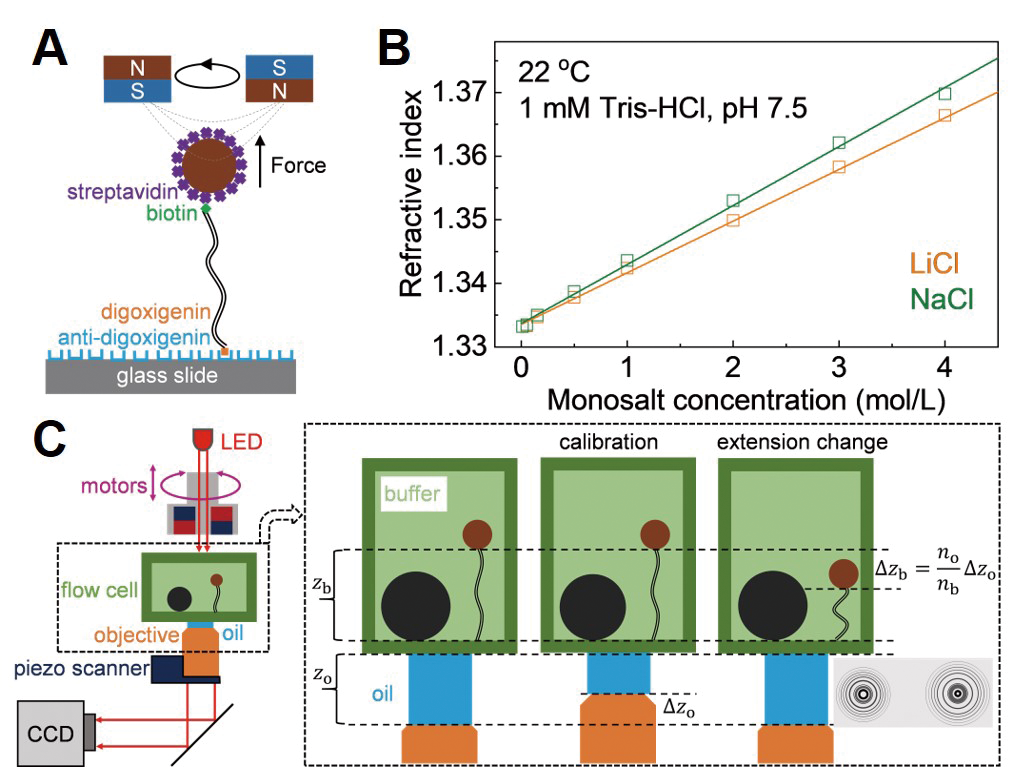


**Figure S2.** Effects of the refractive index of the buffer (n_b_) at different salt conditions. (A) Tethering of a DNA or RNA molecule between a glass slide and a para-paramagnetic bead in MT experiments. (B) The measured n_b_ as a function of salt concentration (c) for LiCl NaCl. Fitting to linear functions yield n_b_ = 1.3336+K*c, where the coefficients K is about 0.00812 for LiCl and 0.00928 for NaCl in the unit of L/mol. (C) How the refractive index of the buffer affected the determination of DNA/RNA extension. Prior to assessing the twist-extension curve for every RNA or DNA molecule under each salt condition, we established a comprehensive library of diffraction patterns for the bead at each vertical objective position utilizing a piezo scanner, which alters the thickness of the objective oil by Δz_o_. The objective oil's refractive index (n_o_) is approximately 1.518 at 22°C. While evaluating the twist-extension curve for every RNA or DNA molecule under a specific salt condition, we ascertained the alteration in the extension of the molecule (Δz_b_) by correlating the bead's diffraction pattern with those in our library. Given that identical diffraction patterns for the bead signified an equal optical length, we derived Δz_b_=(n_o_/n_b_)Δz_o_. Hence, the change in the absolute extension of every molecule caused by the change of force was determined.


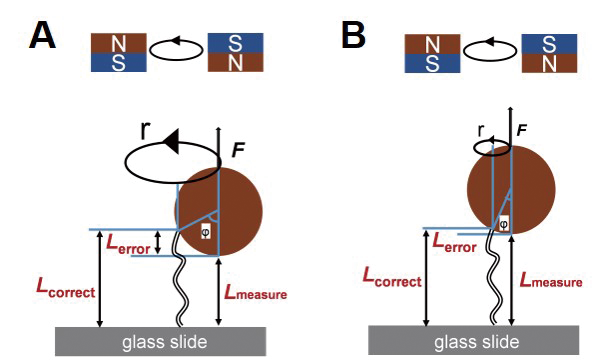


**Figure S3.** Selection of the paramagnetic beads. (A)The paramagnetic beads discarded. If a DNA or RNA molecule anchored far away from the center bottom of a paramagnetic bead, an error (*L*_error_) occurs between the measured extension (*L*_measure_) and the correct extension (*L*_correct_) of the molecule. The value of *L*_error_ can be calculated using the equation $L_{\text{error}}=R_{\text{bead}}-\sqrt{R_{\text{bead}}^{2}-R_{\text{rotation}}^{2}}$, where $R_{\text{bead}}$ represents the radius of the paramagnetic bead (0.5 μm) and R_"rotation" represents the radius of the rotation circle around the molecule-bead anchor point, which changes with the applied force (1). Since $R_{\text{rotation}}$ changes with force, *L*_error_ also changes with force, resulting in a distortion of the correct force-extension of the molecule. Any paramagnetic beads with $R_{\text{rotation}}$ larger than 100 nm at 1 pN were discarded. (B) The paramagnetic beads selected. If a DNA or RNA molecule is anchored near the center bottom of a paramagnetic bead, $L_{\text{error}}$ is negligible. The error *L*_error_ <10 nm if $R_{\text{rotation}}$<100 nm.


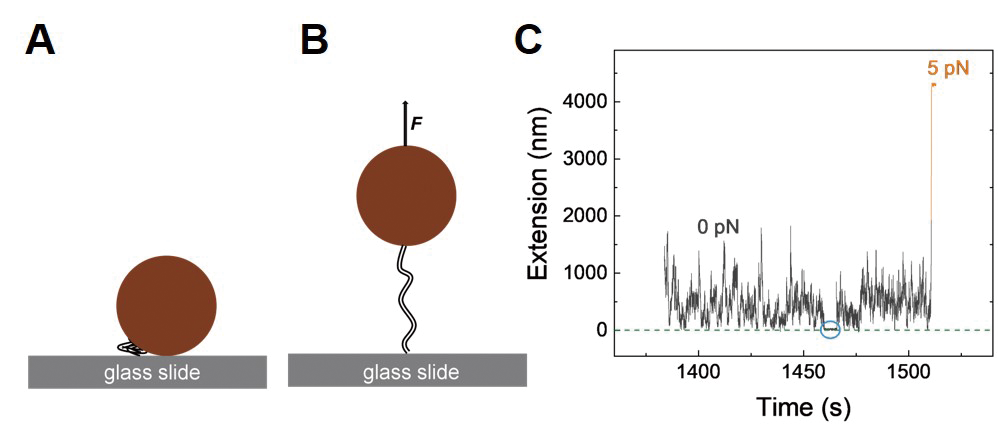


**Figure S4.** Determining the glass top's height. We performed this experiment at 500 mM NaCl. (A) When the magnets are far away, the paramagnetic beads dropped down to the top of the functionalized glass surface. (B) When the magnetic is near, the paramagnetic beads are attracted and the tethered DNA or RNA molecule is stretched with force. (C) At zero force, the paramagnetic beads can contact with and sometimes be temporarily absorbed to the glass top (cycle circle), where the glass top's height is determined. The extension of the molecule is set to zero when the paramagnetic beads contact the glass top.


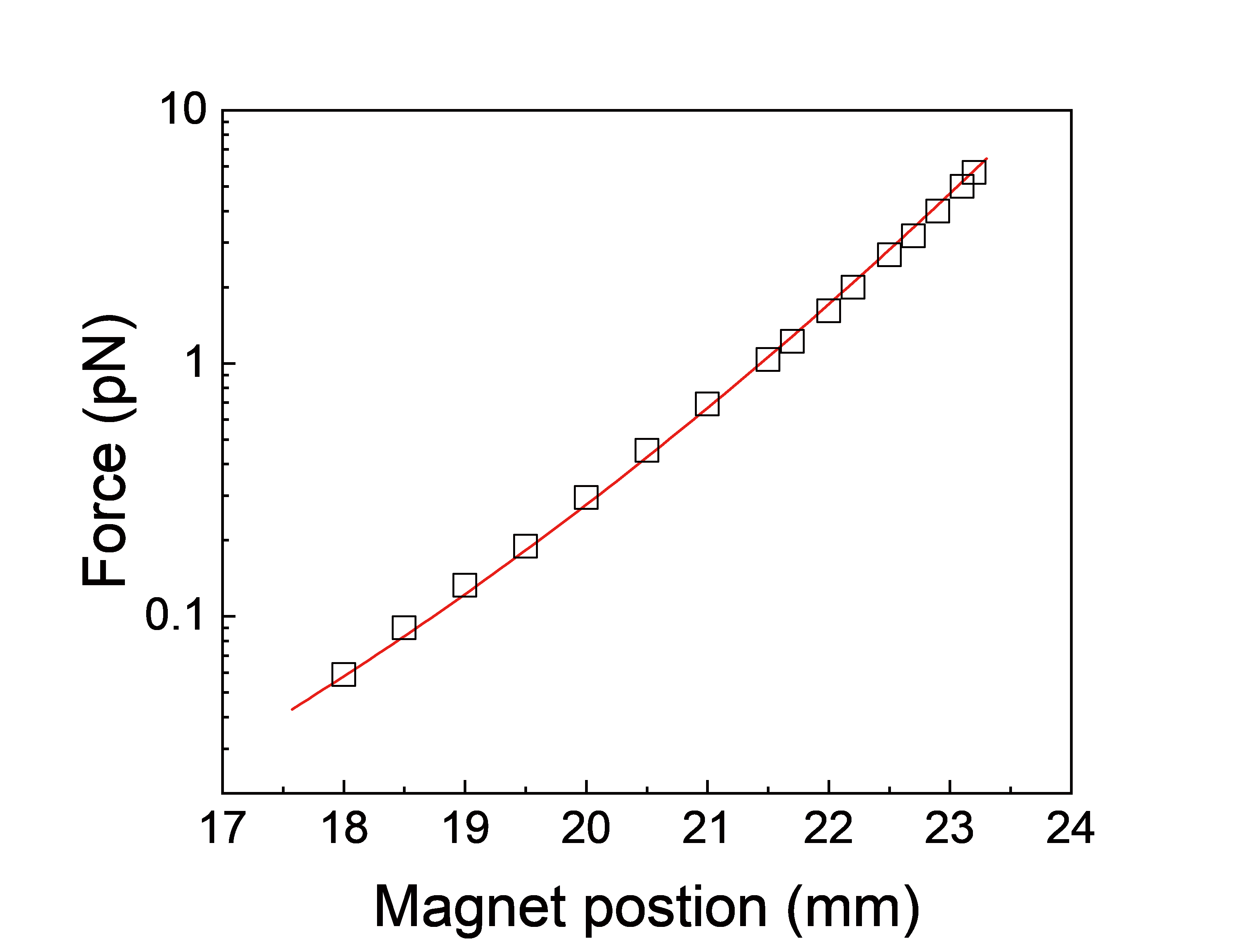


**Figure S5.** Determining the applied force. For each selected paramagnetic bead tethered to a DNA or RNA molecule, we measured the applied forces (*F*) at a series of positions of the magnets (*h*) and fitted them to the function *F*=exp(a*h*^2^+b*h*+c). Subsequently, we calculated the applied force using the function for force-extension curve measurements at different salt concentrations with the same molecule. In the figure illustrating the determination of the function *F*(*h*) for a paramagnetic bead, the coefficients are a = 0.02843, b = -0.31071, and c = -6.40713. We performed the experiment at 500 mM NaCl.


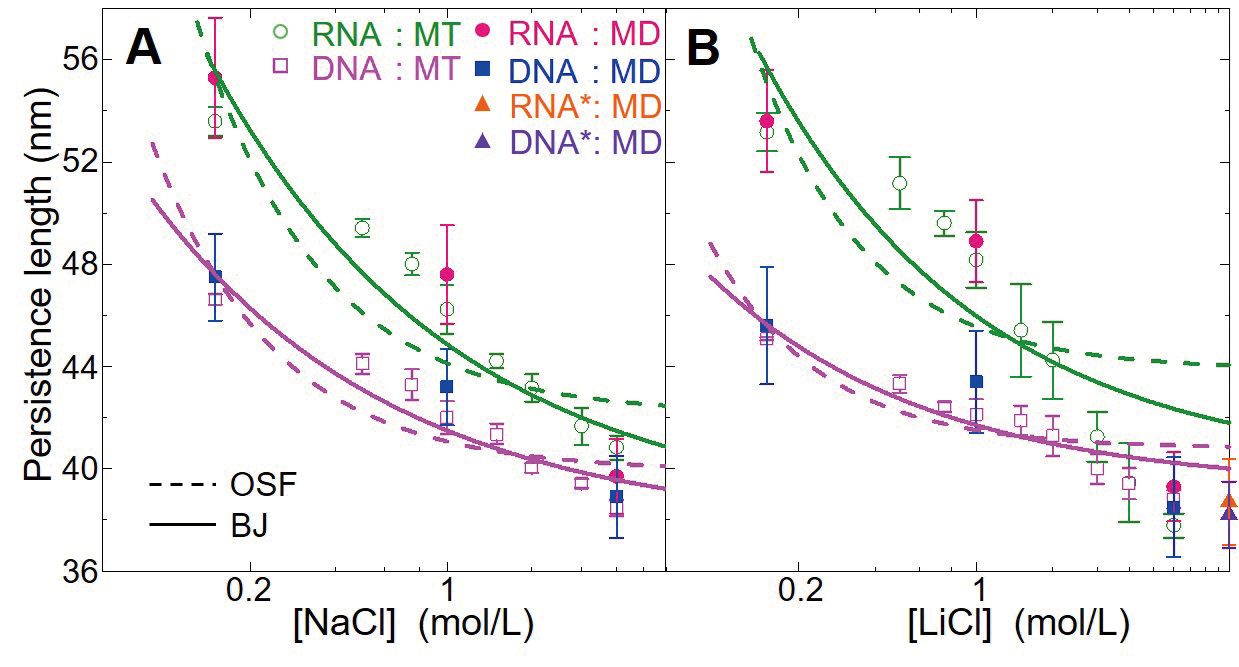


**Figure S6.** Persistence length *P* for dsRNA and dsDNA as a function of monovalent salt concentration. To describe the salt dependence of *P*, we fitted two theoretical models to our experimental data: *P* = *P*_nel_ + C/[salt] from the OSF model developed by Odijk and independently by Skolnick and Fixman, and *P* = *P*_nel_ + C/[salt]^0.5^ from the BJ model developed by Barrat and Joanny. Here, [salt] is monovalent salt concentration and *P*_nel_ is the non-electrostatic persistence length. *P*_nel_ and C are treated as two fitting parameters, and the fitting parameters are list in Table S2.


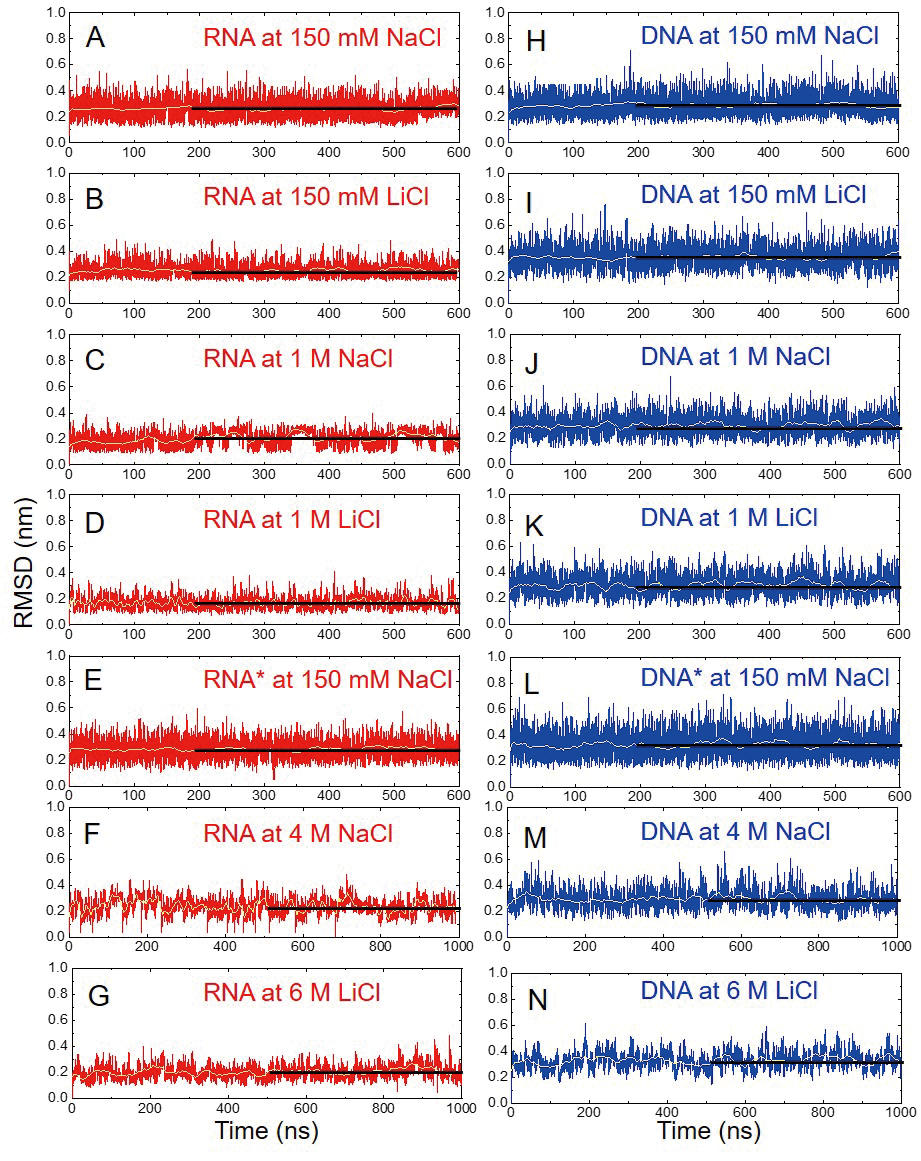


**Figure S7.** The root-mean-square deviation (RMSD) versus MD running time for the central 14-bp segment of the dsRNA (left) and dsDNA (right) duplexes. The light lines represent the RMSD values averaged over every 2 ns. The straight lines denote the averaged RMSD values in the range of 200-600 ns for ordinary salts/“neutral” dsRNA*/dsDNA* and 500-1000 ns for ultra-high salts, respectively.


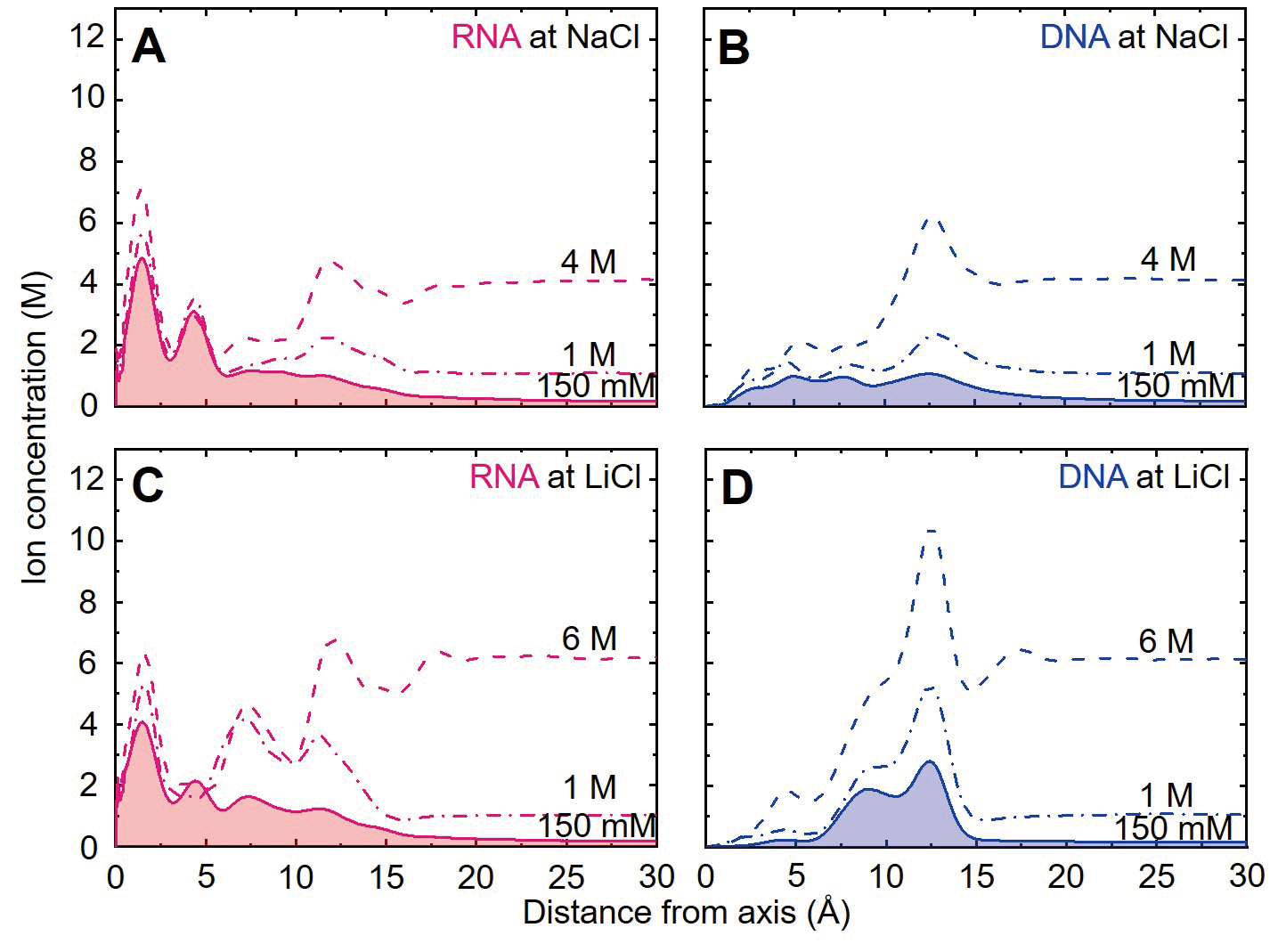


**Figure S8.** The radial concentration distributions of Na^+^ and Li^+^ around 20-bp dsRNA and dsDNA obtained from our MD simulations. At a large radial distance, the Na^+^ and Li^+^ concentrations converge to our desired concentrations (150 mM NaCl, 150 mM LiCl, 1 M NaCl, 1 M LiCl, 4 M NaCl, and 6 M LiCl).


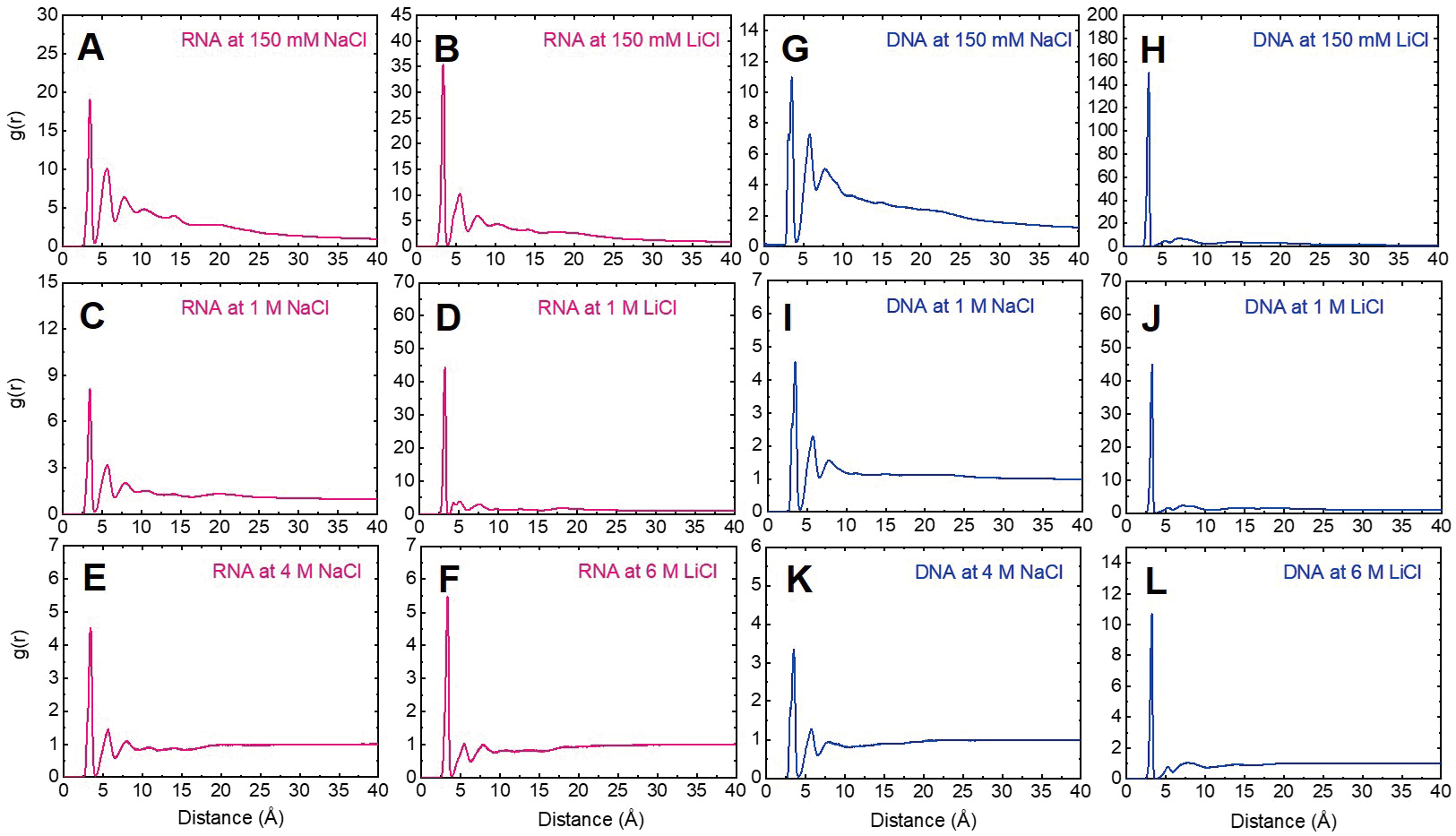


**Figure S9.** The radial distribution functions, g(*r*), of Na^+^ and Li^+^ ions around phosphates of the dsRNA and dsDNA at different ionic concentrations from our MD simulations.


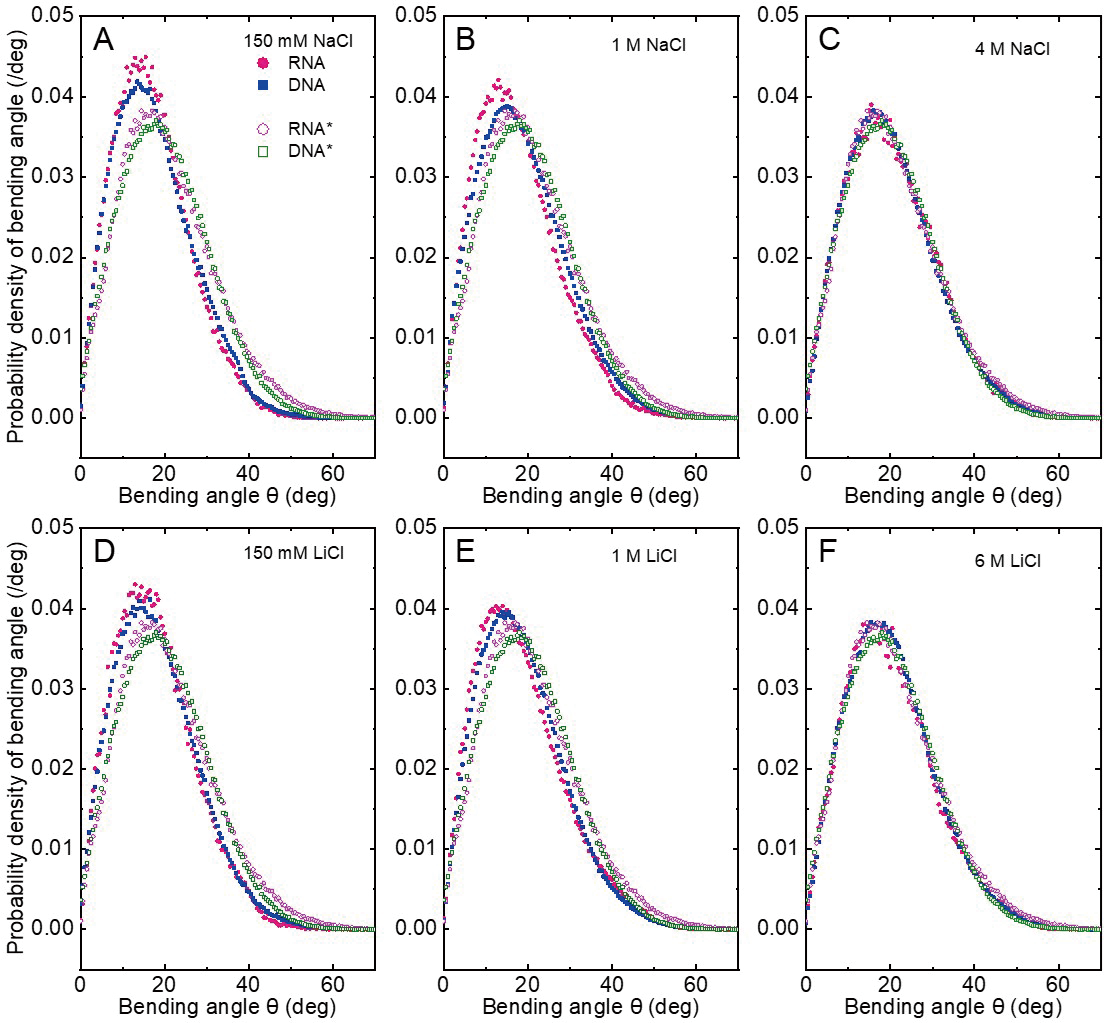


**Figure S10.** The bending angle distributions *p*(*θ*) versus bending angle *θ* over dsRNA and dsDNA segments. RNA* and DNA* denote the electrically “neutral” dsRNA and dsDNA. Here, for convenience, the segments with 13-bp dsRNA and 11-bp dsDNA, which have a similar contour length of ~3.3 nm, were used in our calculations for dsRNA and dsDNA, respectively. The bending persistence length *P* of dsRNA and dsDNA can be calculated by fitting *p*(*θ*) according to Eq. (2) in the main text.


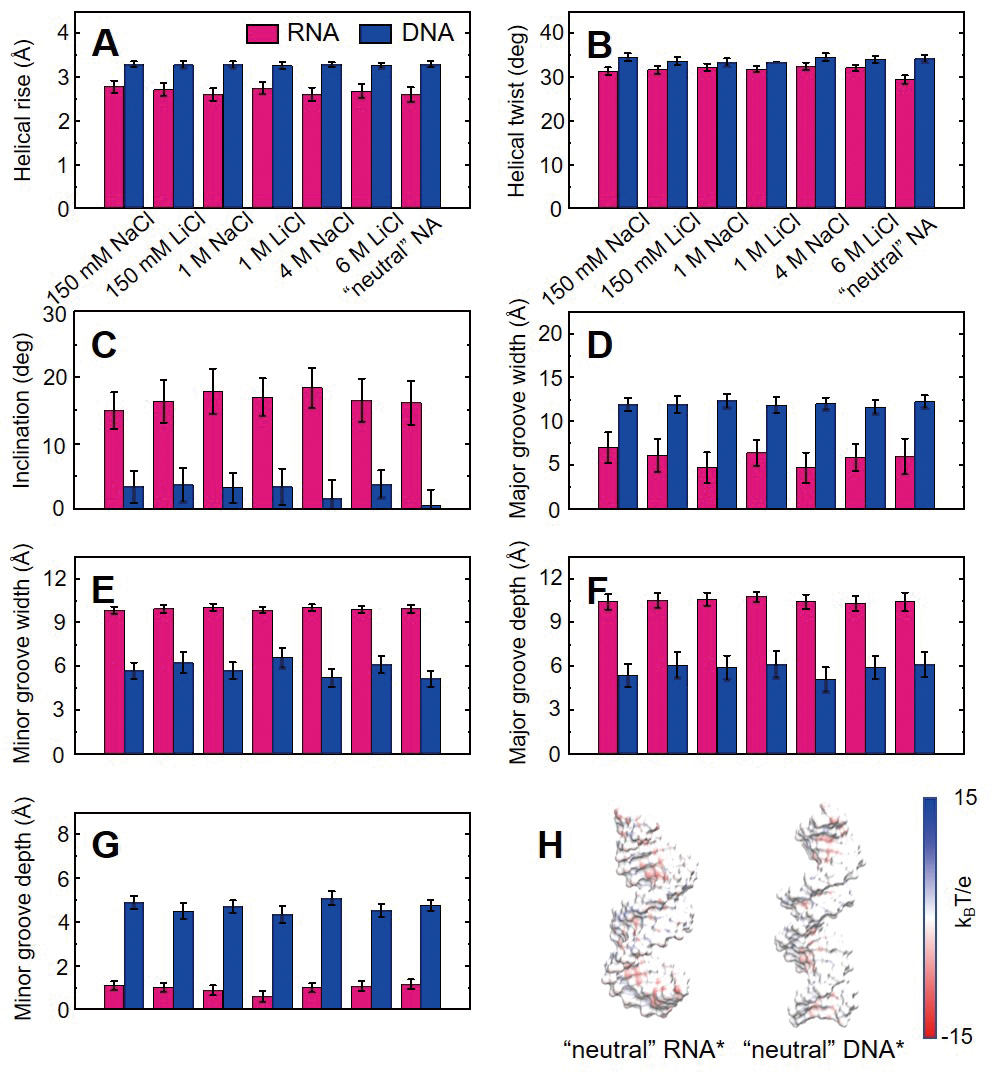


**Figure S11.** The helical parameters of the central 14-bp segments for the dsRNA and dsDNA at different ion conditions and for dsRNA* and dsDNA*, including helical rise (A), helical twist (B), inclination (C), major groove width (D), minor groove width (E), major groove depth (F), and minor groove depth (G). The error bars are the standard deviations to the respective mean values. (H) Representative structures of dsRNA* (left) and dsDNA* (right) showing the surface electrostatic potentials by the PB solver of APBS.


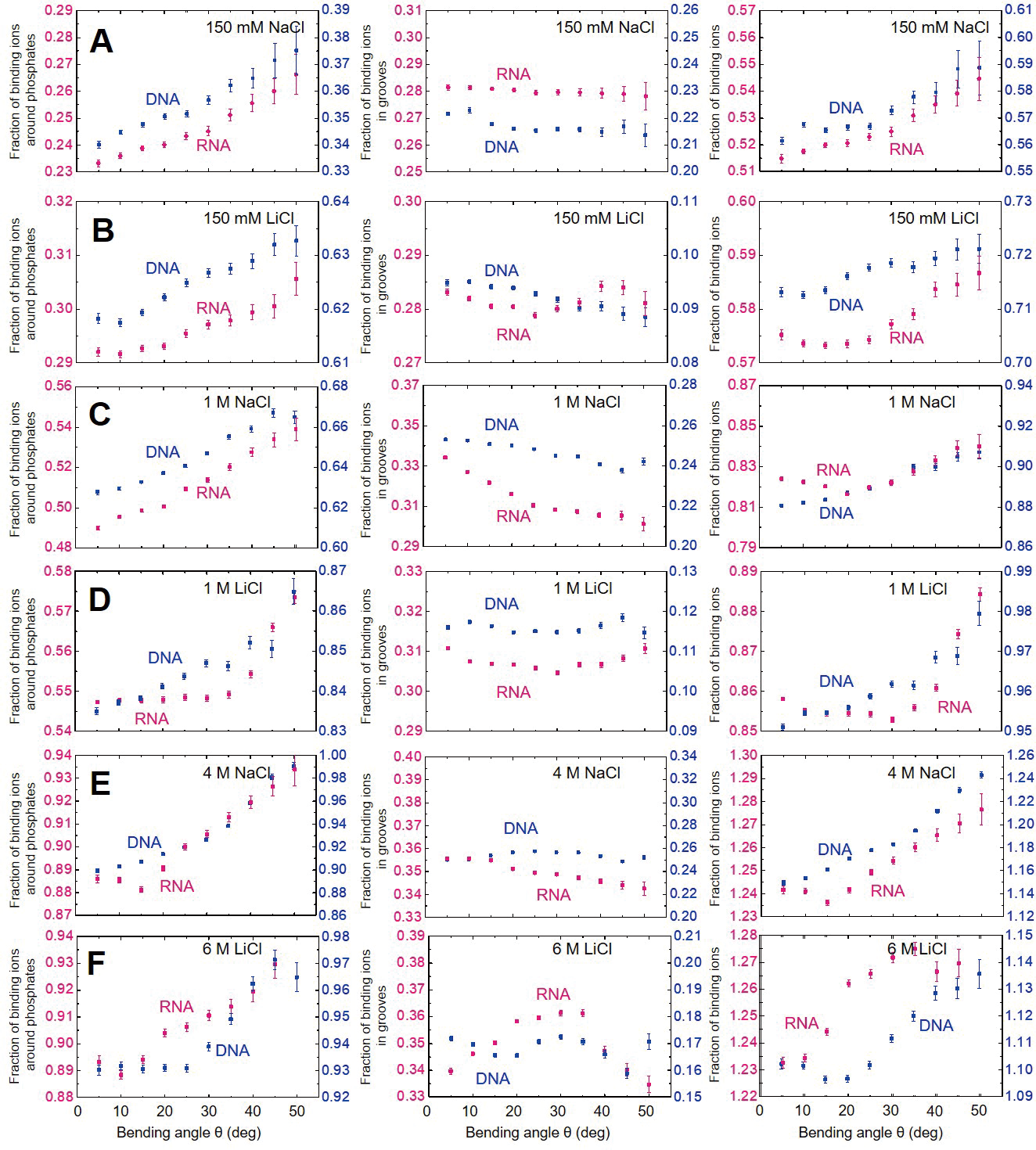


**Figure S12.** (A-F) Average charge fractions of external, internal, and total binding ions as functions of bending angle for the dsRNA (red) and dsDNA (blue) at different ionic conditions.


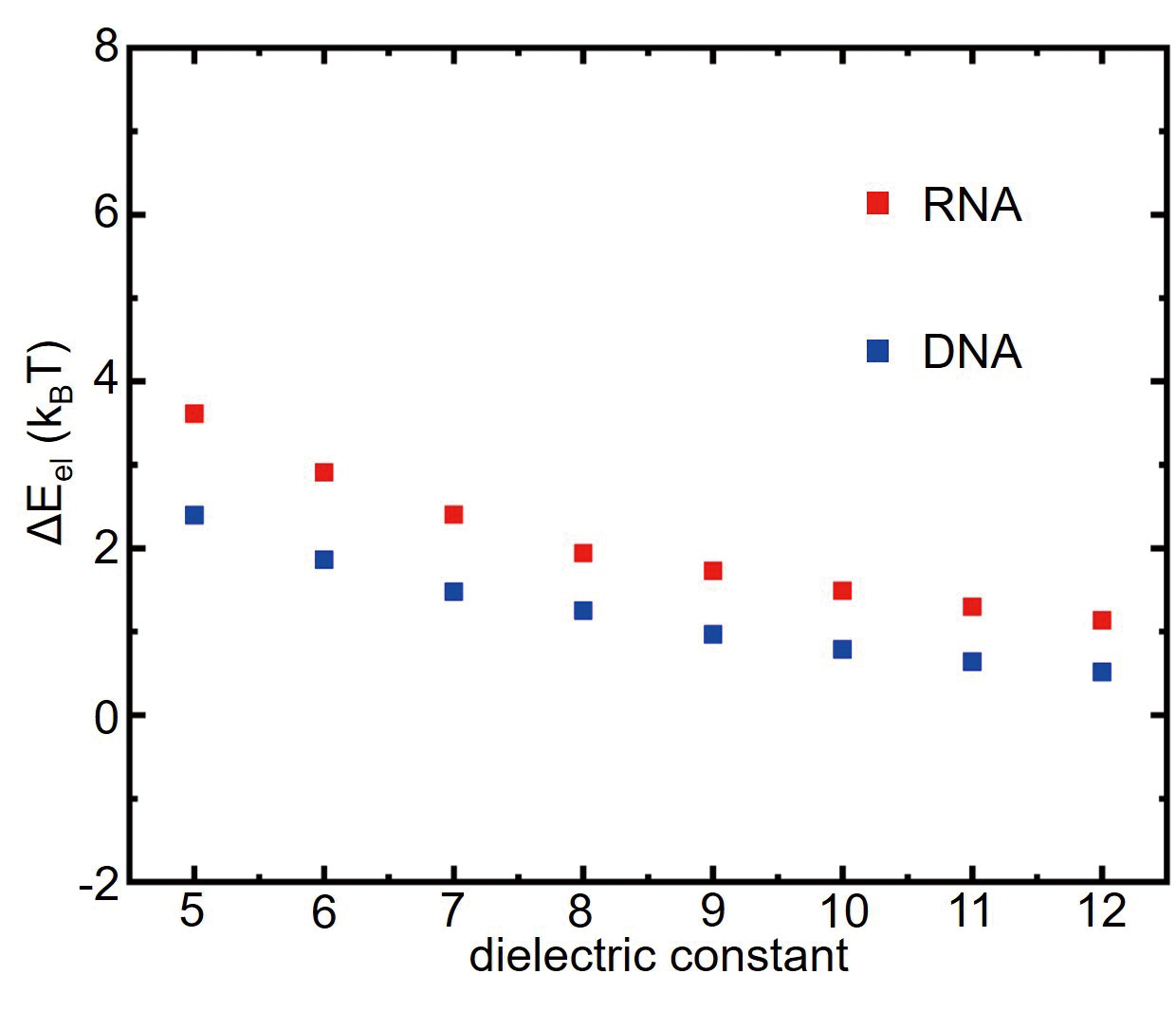


**Figure S13.** The electrostatic bending energy Δ*E*_el_ versus the dielectric constant of dsRNA and dsDNA for bending angle 50˚ over a 13-bp dsRNA/11-bp dsDNA length. Here, Δ*E*_el_ was calculated through the Poisson-Boltzmann theory with the APBS (12) for the conformations with bending angles 0˚ and 50˚ from the MD simulations at 150 mM NaCl.


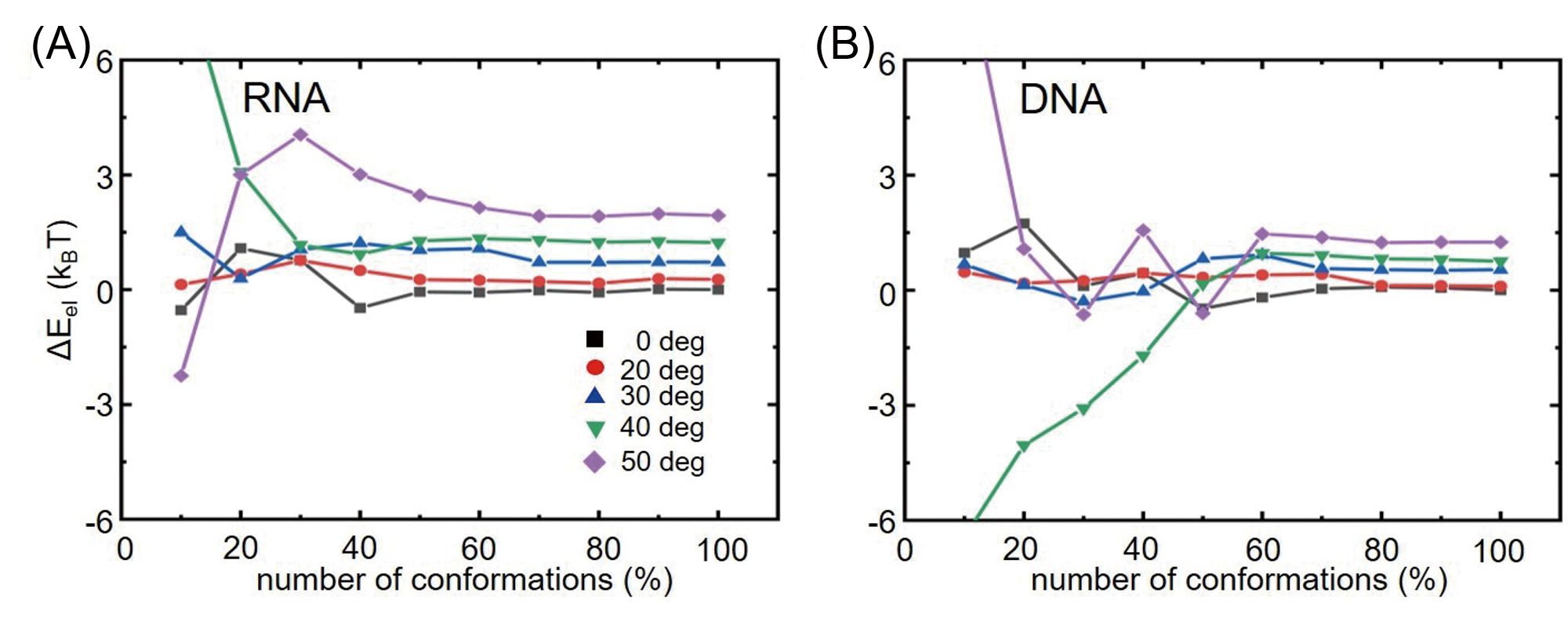


**Figure S14.** The calculated electrostatic bending energy Δ*E*_el_ for different bending angles (0˚, 20˚, 30˚, 40˚ and 50˚) of a 13-bp dsRNA (left) and a 11-bp dsDNA (right) segment versus the number of conformations used in the PB calculations from the MD simulations at 150 mM NaCl. The total numbers used in the PB calculations for different bending angles are listed in Table S6.


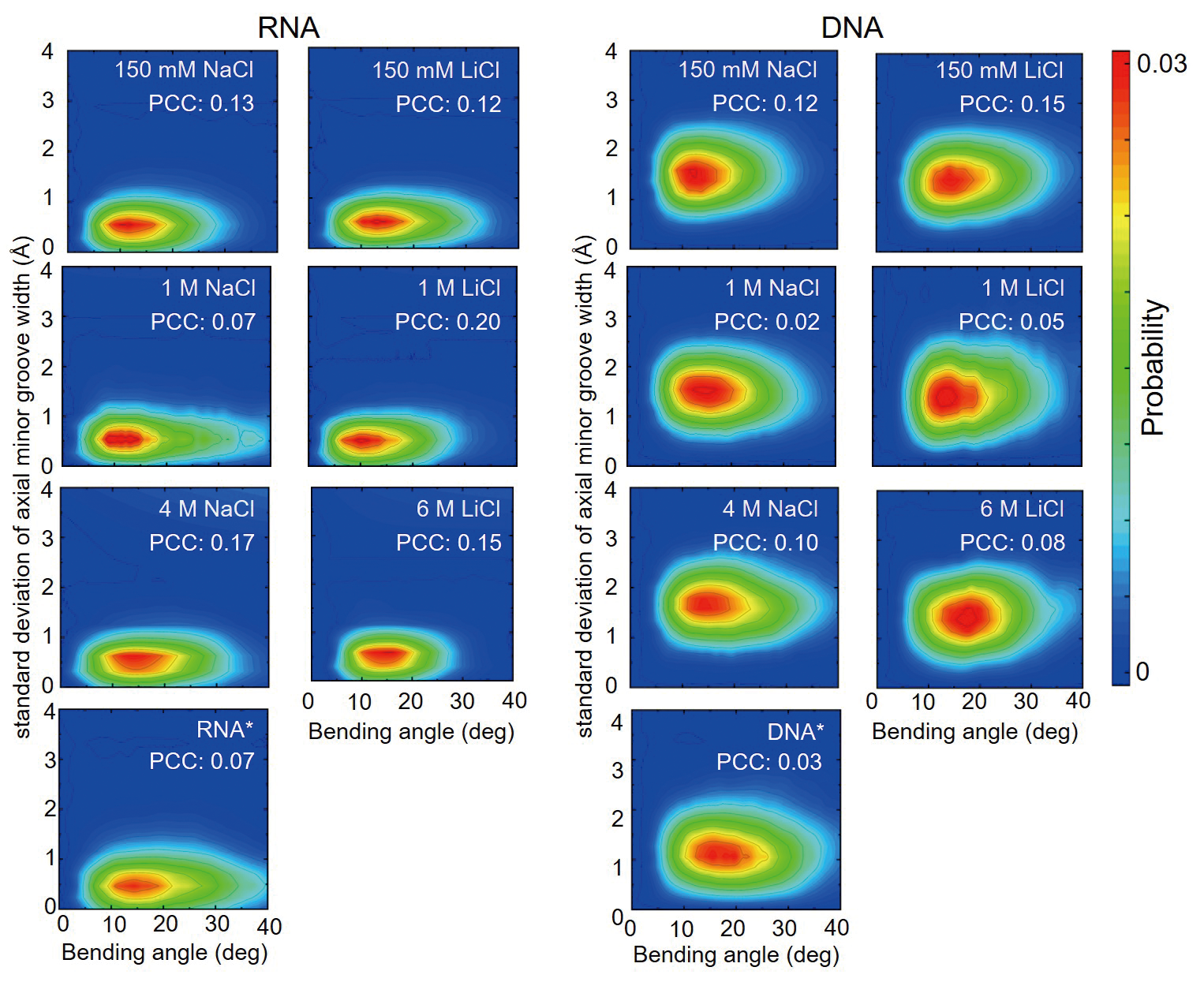


**Figure S15.** Relationships between bending angle and standard deviation of minor groove width from the all-atom MD simulations at different ionic conditions for dsRNA and dsDNA. RNA* and DNA* denote the electrically “neutral” dsRNA and dsDNA. Here, PCCs stand for the Pearson correlation coefficients.


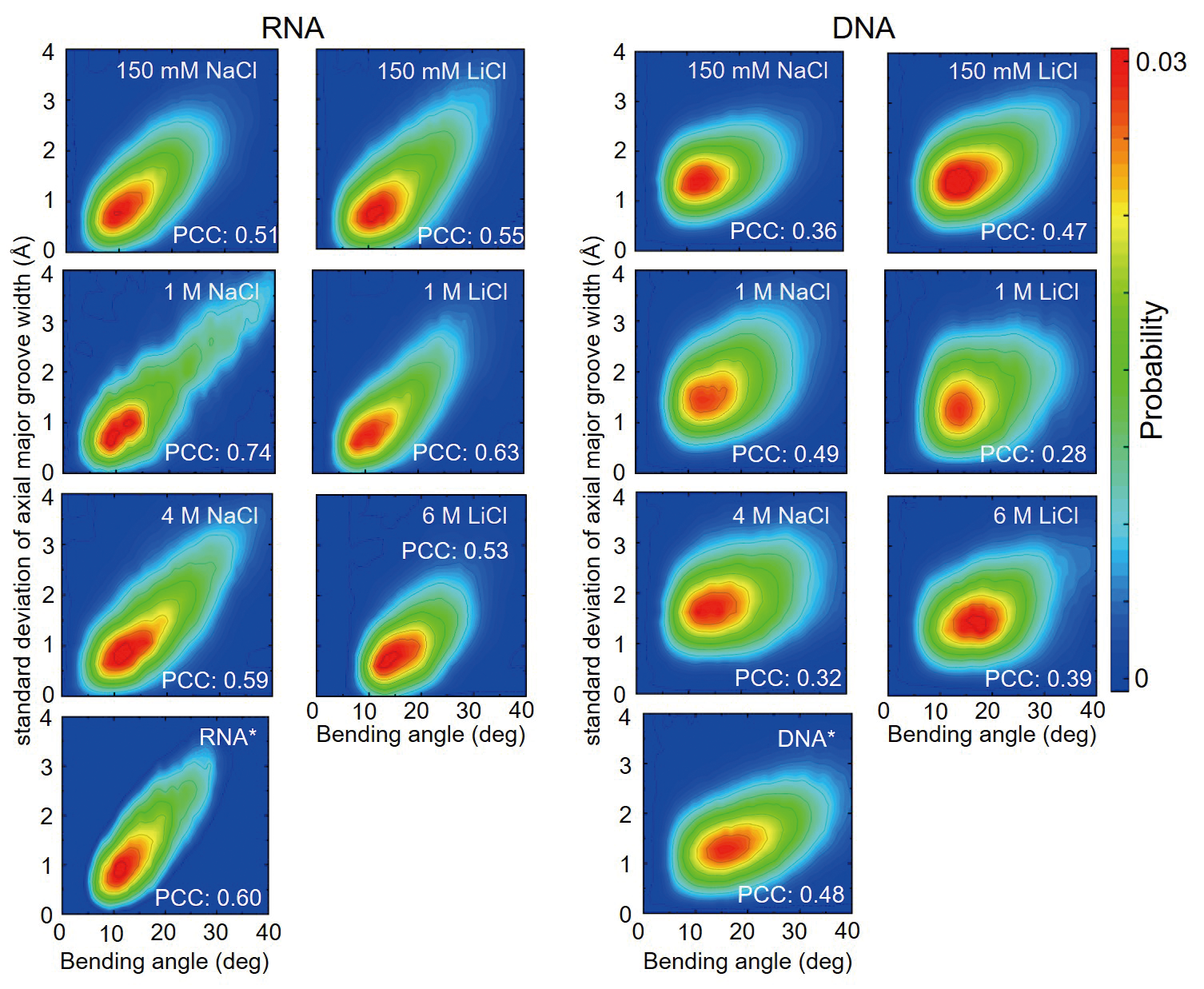


**Figure S16.** Relationships between bending angle and standard deviation of major groove width from the all-atom MD simulations at different ionic conditions for dsRNA and dsDNA. RNA* and DNA* denote the electrically “neutral” dsRNA and dsDNA. Here, PCCs stand for the Pearson correlation coefficients.


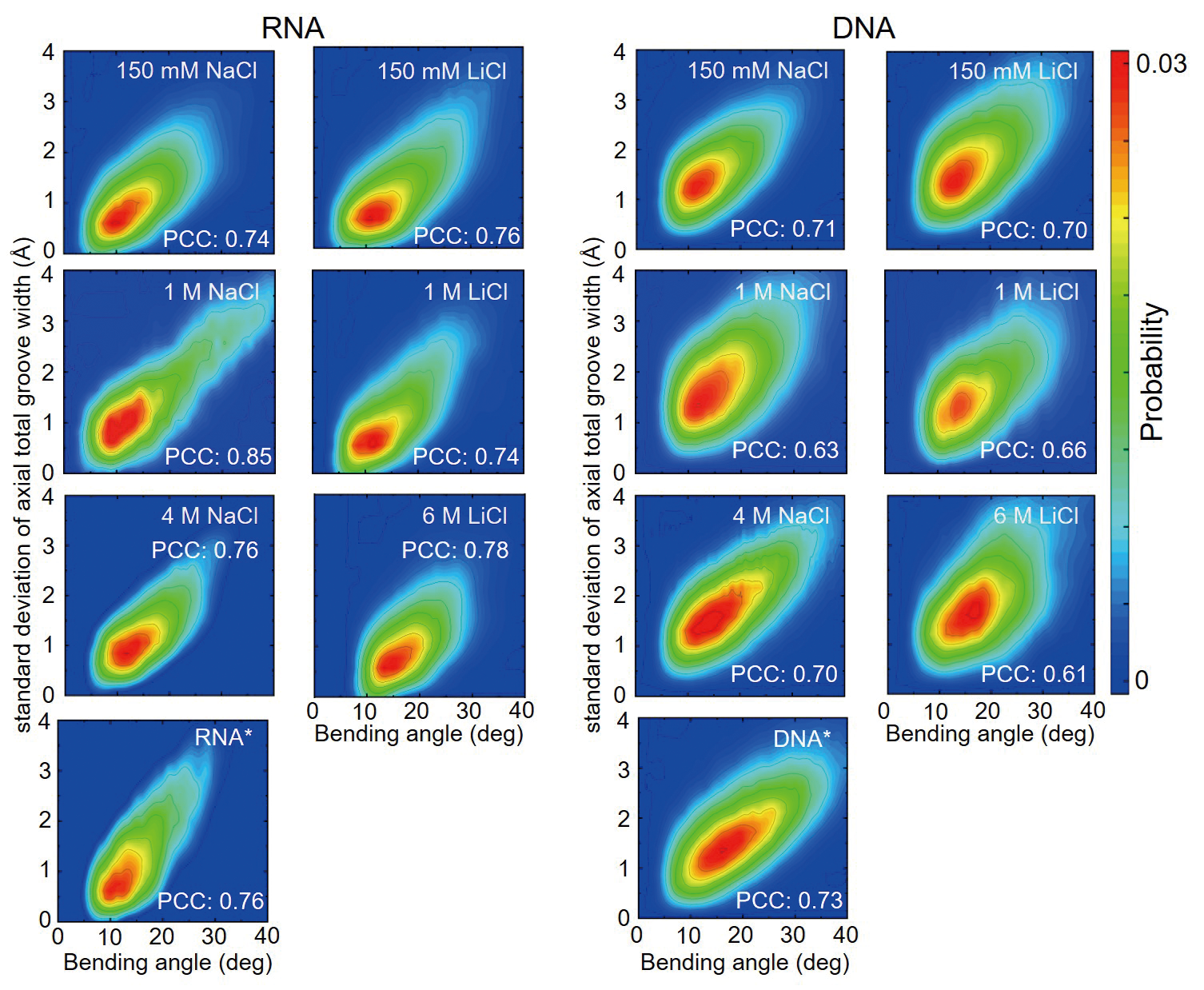


**Figure S17.** Relationships between bending angle and standard deviation of total groove width from the all-atom MD simulations at different ionic conditions for dsRNA and dsDNA. RNA* and DNA* denote the electrically “neutral” dsRNA and dsDNA. Here, PCCs stand for the Pearson correlation coefficients.


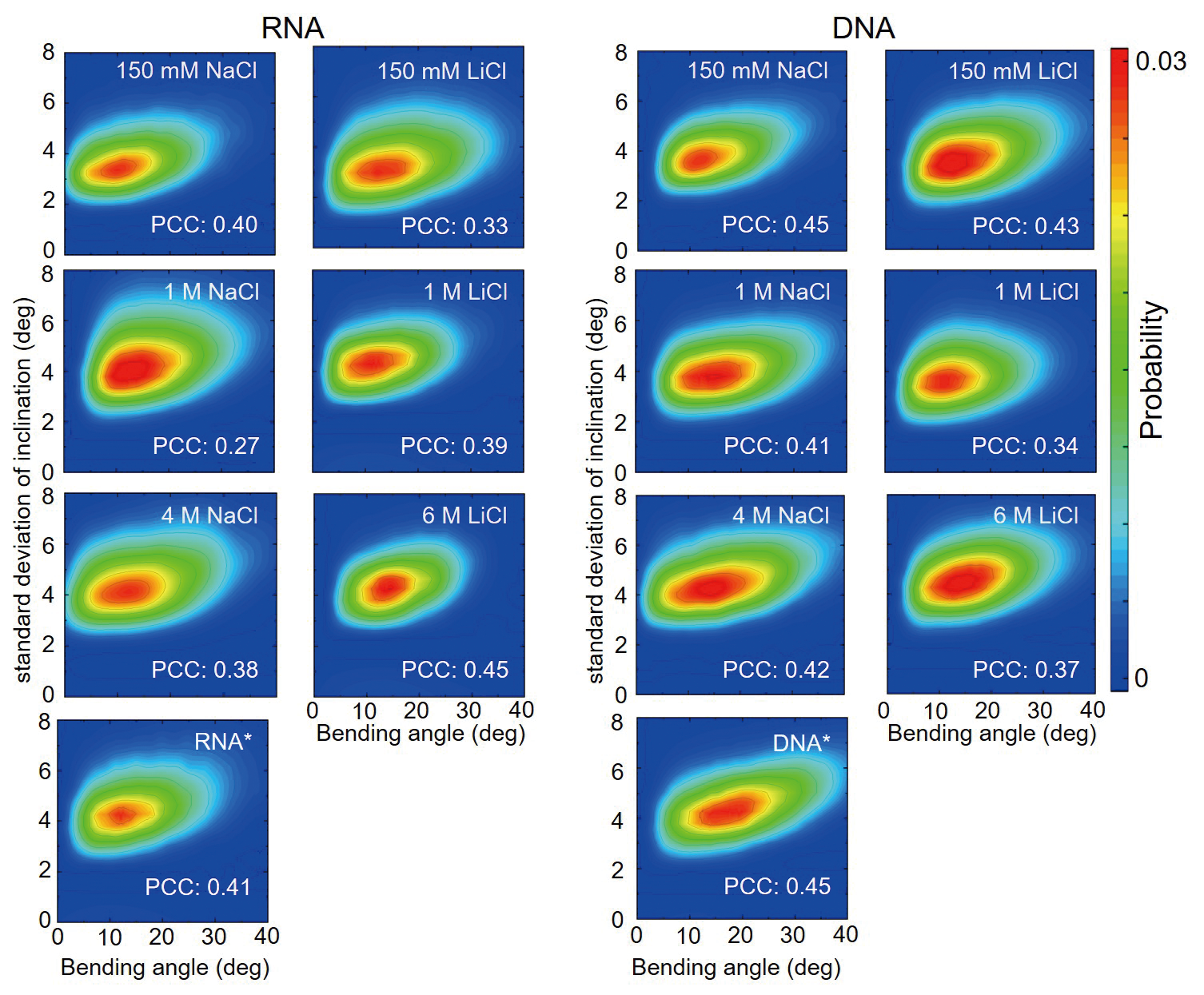


**Figure S18.** Relationships between bending angle and standard deviation of base-pair inclination along dsRNA and dsDNA from the all-atom MD simulations at different ionic conditions. RNA* and DNA* denote the electrically “neutral” dsRNA and dsDNA. Here, PCCs stand for the Pearson correlation coefficients.


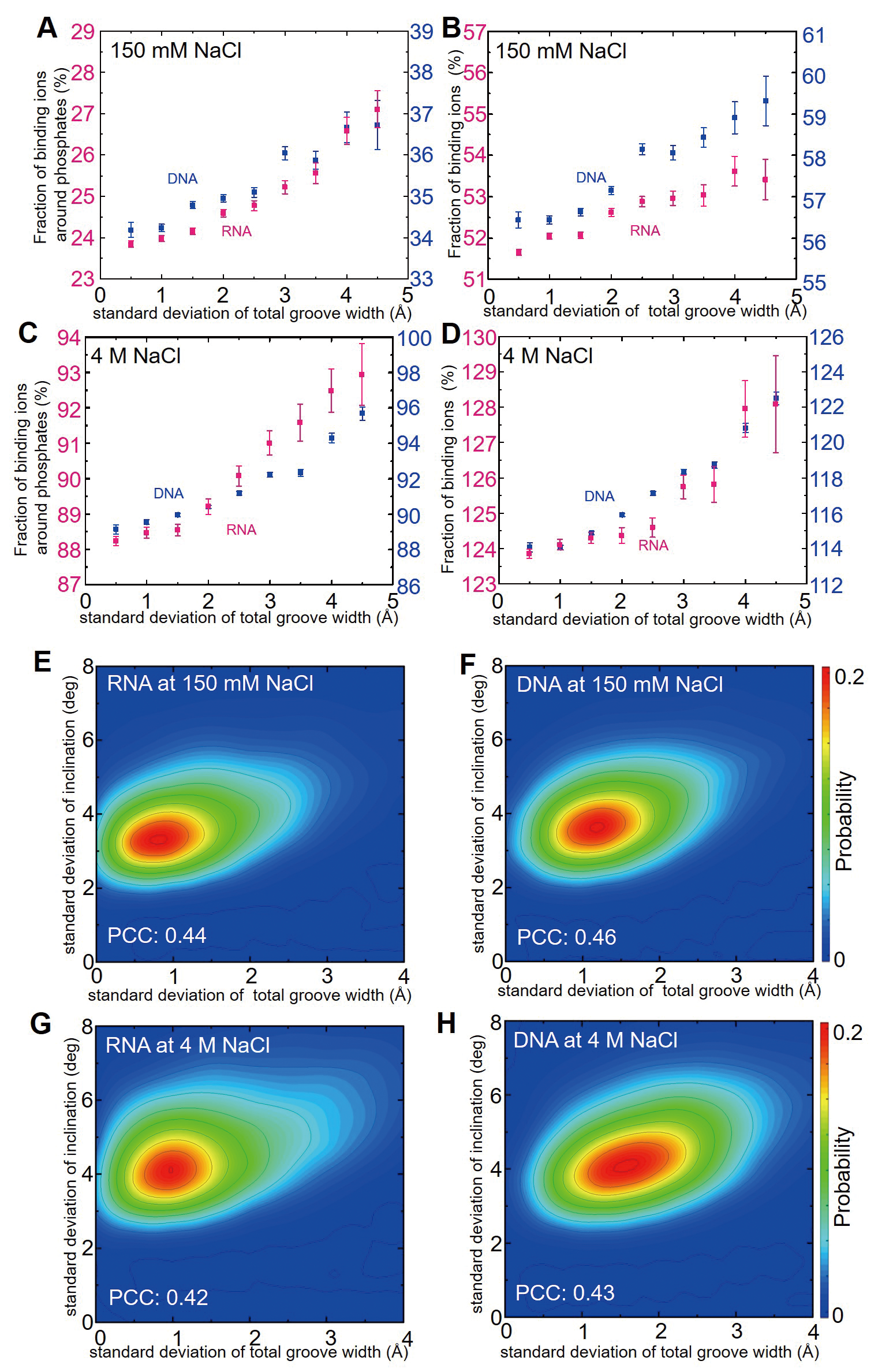


**Figure S19.** (A-D) Average charge fractions of external and total binding ions for dsRNA (red) and dsDNA (blue) structures as functions of bending angle. (E-H) The relationships between standard deviation of total groove width and standard deviation of base pair inclination for dsRNA and dsDNA structures at 150 mM and 4 M NaCl.


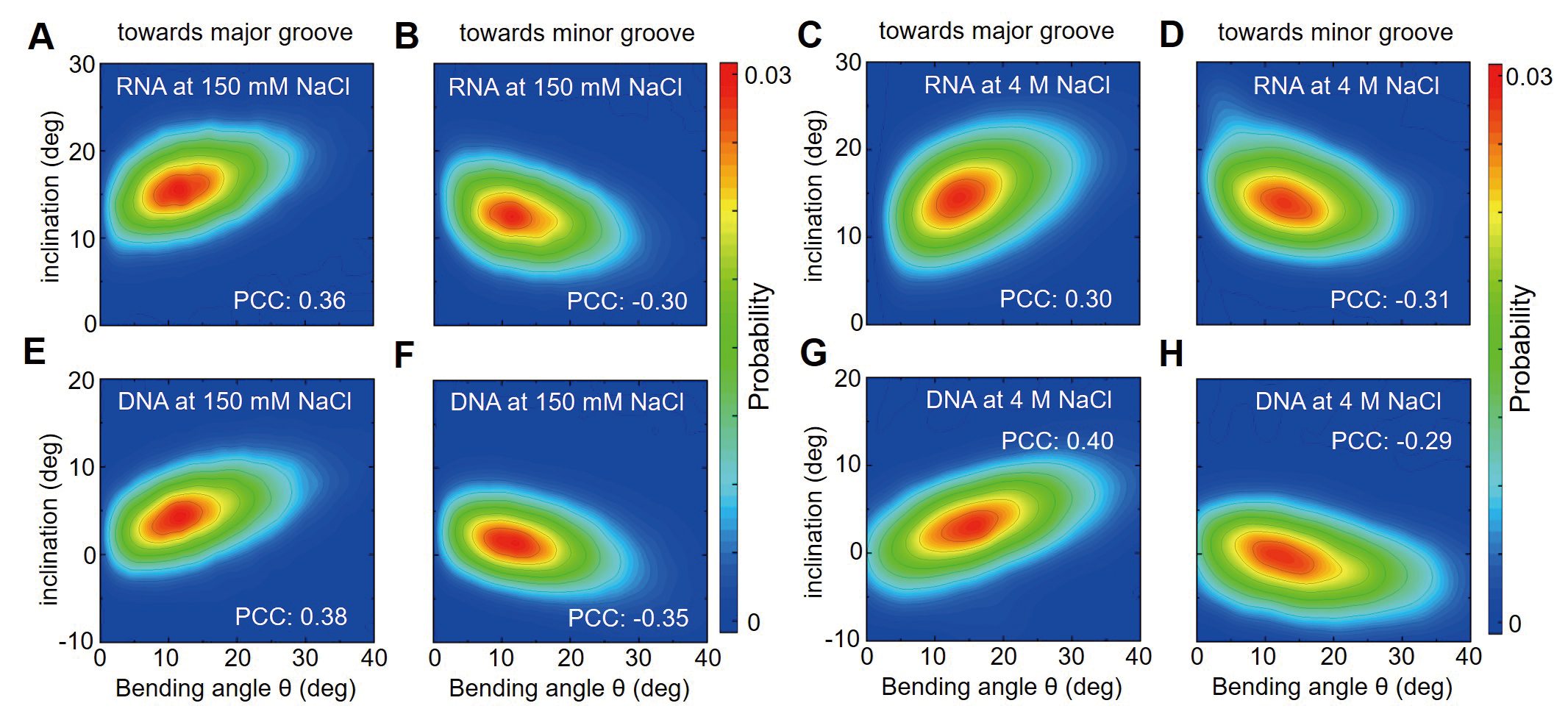


**Figure S20.** The relationships between bending angle and base-pair inclination for dsRNA (A-D) and dsDNA (E-H) at 150 mM NaCl and 4 M NaCl. Bending around the central base pair is classified into that towards major groove and that towards minor groove. The inclination was averaged over those of the central three base pairs to clearly distinguish the bending direction (major groove or minor groove) (18). The figure indicates that, bending towards major grooves generally leads to an increase in inclination, while bending towards minor grooves generally leads to a decrease.

**Table S1.** The persistence lengths P of dsRNA and dsDNA at typical NaCl and LiCl concentrations from our MT experiments and MD simulations.*

| Ion concentration (M) | *P* for dsRNA (nm) | | *P* for dsDNA (nm) | |
| --- | --- | --- | --- | --- |
|  | NaCl | LiCl | NaCl | LiCl |
| 0.15 | 53.59±0.57 (55.31±2.35) | 53.17±0.75 (53.58±1.98) | 46.61±0.20 (47.51±1.70) | 45.09±0.06 (45.63±2.31) |
| 0.5 | 49.42±0.35 | 51.17±1.01 | 44.11±0.39 | 43.32±0.34 |
| 0.75 | 48.01±0.45 | 49.61±0.49 | 43.28±0.60 | 42.42±0.18 |
| 1 | 46.23±0.97 (47.63±1.92) | 48.17±1.08 (48.92±1.64) | 42.00±0.64 (43.21±1.51) | 42.14±0.59 (43.37±2.00) |
| 1.5 | 44.21±0.28 | 45.42±1.81 | 41.36±0.41 | 41.89±0.58 |
| 2 | 43.17±0.53 | 44.25±1.51 | 40.03±0.18 | 41.28±0.80 |
| 3 | 41.67±0.73 | 41.25±0.97 | 39.41±0.18 | 39.99±0.59 |
| 4 | 40.83±0.47 (39.70±1.45) | 39.46±1.54 | 38.48±0.32 (38.88±1.60) | 39.41±0.60 |
| 6 |  | 37.78±0.47 (39.30±1.35) |  | 38.81±0.36 (38.51±1.95) |
| neutral | (38.73±1.68) |  | (38.19±1.30) |  |

*The values in parenthesis denote those from the MD simulations.

**Table S2.** The fitting parameters of the OSF and BJ models for the experimental data of persistence length *P* from our MT measurements.

| model | dsNA | Ionic condition | Free parameters | |
| --- | --- | --- | --- | --- |
|  |  |  | *P*_nel_ (nm) | *C* (nm•M) |
| OSF | dsRNA | NaCl | 42.13 | 1.98 |
|  |  | LiCl | 43.87 | 1.68 |
|  | dsDNA | NaCl | 39.91 | 1.15 |
|  |  | LiCl | 40.79 | 0.72 |
| BJ | dsRNA | NaCl | 38.10 | 6.77 |
|  |  | LiCl | 38.74 | 6.75 |
|  | dsDNA | NaCl | 37.73 | 3.86 |
|  |  | LiCl | 39.31 | 2.49 |

**Table S3.** The atom charges of the phosphate groups for 5′ end residues, 3′ end residues, and the central residues for the electrically “neutral” dsRNA and dsDNA.

| Residue | Atom type | AMBER ff99bsc1+χ_OL3_ | | After modification | |
| --- | --- | --- | --- | --- | --- |
|  |  | DNA | RNA | DNA* | RNA* |
| 5′ end | O5′ | -0.6318 | -0.6223 | -0.6318 | -0.6223 |
|  | O3′ | -0.5232 | -0.5246 | -0.1508 | -0.1523 |
| central | O5′ | -0.4954 | -0.4989 | -0.1428 | -0.1448 |
|  | P | 1.1659 | 1.1662 | 0.3360 | 0.3386 |
|  | OP1/OP2 | -0.7761 | -0.7760 | -0.2236 | -0.2253 |
|  | O3′ | -0.5232 | -0.5246 | -0.1508 | -0.1523 |
| 3′ end | O5′ | -0.4954 | -0.4989 | -0.1428 | -0.1448 |
|  | P | 1.1659 | 1.1662 | 0.3360 | 0.3386 |
|  | OP1/OP2 | -0.7761 | -0.7760 | -0.2236 | -0.2253 |
|  | O3′ | -0.6549 | -0.6541 | -0.6549 | -0.6541 |

**Table S4.** The charge fractions of binding cations (over anions) per nucleotide from our MD simulations at different ion conditions for dsRNA and dsDNA.

|  | dsRNA | | | | | | dsDNA | | | | | | |
| --- | --- | --- | --- | --- | --- | --- | --- | --- | --- | --- | --- | --- | --- |
|  | 150 mM | | 1 M | | 4/6 M | | | 150 mM | | 1 M | | 4/6 M | |
|  | NaCl | LiCl | NaCl | LiCl | NaCl | LiCl | | NaCl | LiCl | NaCl | LiCl | NaCl | LiCl |
| Phosphates | 0.240 | 0.292 | 0.498 | 0.548 | 0.887 | 0.900 | | 0.356 | 0.622 | 0.640 | 0.840 | 0.914 | 0.929 |
| Major groove | 0.249 | 0.234 | 0.272 | 0.243 | 0.299 | 0.270 | | 0.130 | 0.060 | 0.161 | 0.078 | 0.145 | 0.096 |
| Minor groove | 0.032 | 0.046 | 0.052 | 0.063 | 0.054 | 0.080 | | 0.085 | 0.034 | 0.087 | 0.040 | 0.106 | 0.067 |

**Table S5.** The bending energies Δ*E*_bend_, electrostatic bending energy Δ*E*_el_, and non-electrostatic bending energy Δ*E*_nel_ for dsRNA and dsDNA at 150 mM NaCl and the bending energies Δ*E*_bend_^*^ for dsRNA* and dsDNA* at bending angle 50°.*

|  | dsRNA | dsDNA | Δ*E*_RNA_-Δ*E*_DNA_ (k_B_T) |
| --- | --- | --- | --- |
| Δ*E*_bend_ (k_B_T) | 6.36 | 5.49 | 0.87 |
| Δ*E*_el_ (k_B_T) | 2.00 | 1.20 | 0.80 |
| Δ*E*_nel_ (k_B_T) | 4.36 | 4.29 | 0.07 |
| Δ*E*_bend_^*^ (k_B_T) | 4.43 | 4.34 | 0.09 |

*The data in this table corresponds to the Figure 6D in the MS.

**Table S6.** Numbers of conformations at different bending angles from the MD simulations for the PB calculations.

| Bending angle (deg) | dsRNA | dsDNA |
| --- | --- | --- |
| 0 | 1920 | 1618 |
| 5 | 4351 | 3767 |
| 10 | 7320 | 6274 |
| 15 | 7847 | 6747 |
| 20 | 6607 | 5976 |
| 25 | 4615 | 4285 |
| 30 | 2647 | 3122 |
| 35 | 2117 | 2406 |
| 40 | 1578 | 1800 |
| 42.5 | 1252 | 1415 |
| 45 | 1043 | 1237 |
| 47.5 | 773 | 854 |
| 50 | 396 | 502 |

**Table S7.** Pearson correlation coefficients between bending angle and 16 base-pair parameters and their fluctuations for dsRNA and dsDNA at 150 mM NaCl and 4 M NaCl.*

| Base pair parameter | dsRNA | | | dsDNA | |
| --- | --- | --- | --- | --- | --- |
|  | mean value | standard deviation | mean value | | standard deviation |
|  |  |  | |  |  |
| Shear | 0.004 (0.016) | 0.015 (0.014) | | -0.003 (-0.030) | 0.002 (0.006) |
| Buckle | 0.007 (0.004) | 0.013 (0.033) | | -0.001 (-0.007) | 0.005 (0.009) |
| Stretch | 0.000 (0.006) | 0.006 (0.011) | | -0.006 (-0.002) | 0.007 (0.009) |
| Propeller | 0.050 (0.095) | 0.037 (0.048) | | 0.029 (0.039) | 0.064 (0.011) |
| Stagger | -0.050 (-0.006) | 0.018 (0.025) | | -0.009 (-0.024) | 0.036 (0.026) |
| Opening | 0.007 (0.010) | 0.010 (0.025) | | -0.011 (-0.044) | 0.009 (-0.034) |
|  |  | | |  | |
| Shift | -0.003 (0.026) | 0.024 (0.016) | | -0.010 (-0.098) | -0.020 (-0.075) |
| Tilt | 0.011 (0.035) | 0.008 (0.006) | | -0.003 (-0.045) | 0.007 (0.000) |
| Slide | -0.006(0.135) | 0.060(0.096) | | -0.045(-0.121) | 0.025(-0.038) |
| Roll | 0.017 (-0.203) | 0.260 (0.236) | | 0.000 (-0.040) | 0.229 (0.253) |
| Rise | 0.069 (0.122) | 0.010 (0.022) | | 0.060 (0.076) | 0.020 (0.039) |
| Twist | -0.050 (-0.101) | 0.025 (0.045) | | -0.070 (-0.158) | 0.020 (-0.012) |
|  |  | | |  | |
| Inclination | 0.010 (-0.095) | 0.402 (0.382) | | 0.009 (-0.034) | 0.453 (0.417) |
| Tip | -0.018 (-0.061) | 0.107(0.075) | | -0.001 (0.019) | 0.150 (0.176) |
| Xdisp | 0.005 (-0.017) | 0.032 (0.097) | | -0.049 (-0.075) | 0.066 (0.032) |
| Ydisp | 0.039 (0.039) | 0.059 (0.043) | | 0.003 (0.118) | 0.090 (0.182) |

*The values in parenthesis denote those for 4 M NaCl.

**REFERENCES**

1. Zhang, C., Fu, H., Yang, Y., Zhou, E., Tan, Z., You, H. and Zhang, X.H. (2019) The mechanical properties of RNA-DNA hybrid duplex stretched by magnetic tweezers. *Biophys. J.*, **116**, 196-204.

2. Yang, Y.J., Dong, H.L., Qiang, X.W., Fu, H., Zhou, E.C., Zhang, C., Yin, L., Chen, X.F., Jia, F.C., Dai, L., Tan, Z.J. and Zhang, X.H. (2020) Cytosine methylation enhances DNA condensation revealed by equilibrium measurements using magnetic tweezers. *J. Am. Chem. Soc.*, **142**, 9203-9209.

3. Xiao, S., Zhu, H., Wang, L. and Liang, H. (2014) DNA conformational flexibility study using phosphate backbone neutralization model. *Soft Matter*, **10**, 1045-1055.

4. Savelyev, A., Materese, C.K. and Papoian, G.A. (2011) Is DNA’s rigidity dominated by electrostatic or nonelectrostatic interactions? *J. Am. Chem. Soc.*, **133**, 19290-19293.

5. Wu, Y.Y., Bao, L., Zhang, X. and Tan, Z.J. (2015) Flexibility of short DNA helices with finite-length effect: From base pairs to tens of base pairs. *J. Chem. Phys*, **142**, 125103

6. Bao, L., Zhang, X., Shi, Y.Z., Wu, Y.Y. and Tan, Z.J. (2017) Understanding the relative flexibility of RNA and DNA duplexes: stretching and twist-stretch coupling. *Biophys. J.*, **112**, 1094-1104.

7. Liu, J.H., Xi, K., Zhang, X., Bao, L., Zhang, X.H. and Tan, Z.J. (2019) Structural flexibility of DNA-RNA hybrid duplex: stretching and twist-stretch coupling. *Biophys. J.*, **117**, 74-86.

8. Qiang, X.W., Zhang, C., Dong, H.L., Tian, F.J., Fu, H., Yang, Y.J., Dai, L., Zhang, X.H. and Tan, Z.J. (2022) Multivalent Cations Reverse the Twist-Stretch Coupling of RNA. *Phys. Rev. Lett.*, **128**, 108103.

9. Fu, H., Zhang, C., Qiang, X.W., Yang, Y.J., Dai, L., Tan, Z.J. and Zhang, X.H. (2020) Opposite effects of high-valent cations on the elasticities of DNA and RNA duplexes revealed by magnetic tweezers. *Phys. Rev. Lett.*, **124**, 058101.

10. Liebl, K., Drsata, T., Lankas, F., Lipfert, J. and Zacharias, M. (2015) Explaining the striking difference in twist-stretch coupling between DNA and RNA: A comparative molecular dynamics analysis. *Nucleic Acids Res.*, **43**, 10143-10156.

11. Lavery, R., Moakher, M., Maddocks, J.H., Petkeviciute, D. and Zakrzewska, K. (2009) Conformational analysis of nucleic acids revisited: Curves+. *Nucleic Acids Res.*, **37**, 5917-5929.

12. Baker, N.A., Sept, D., Joseph, S., Holst, M.J. and McCammon, J.A. (2001) Electrostatics of nanosystems: application to microtubules and the ribosome. *Proc. Natl. Acad. Sci. U. S. A.*, **98**, 10037-10041.

13. Baker, N.A. (2004), Poisson–Boltzmann methods for biomolecular electrostatics. *Methods in enzymology*. *Methods in enzymology*. Elsevier, Vol. 383, pp. 94-118.

14. Kuhlman, B. and Baker, D. (2000) Native protein sequences are close to optimal for their structures. *Proc. Natl. Acad. Sci. U. S. A.*, **97**, 10383-10388.

15. Cuervo, A., Dans, P.D., Carrascosa, J.L., Orozco, M., Gomila, G. and Fumagalli, L. (2014) Direct measurement of the dielectric polarization properties of DNA. *Proc. Natl. Acad. Sci. U. S. A.*, **111**, E3624-E3630.

16. Mohsen-Nia, M., Amiri, H. and Jazi, B. (2010) Dielectric constants of water, methanol, ethanol, butanol and acetone: measurement and computational study. *J. Solution Chem.*, **39**, 701-708.

17. Dolinsky, T.J., Nielsen, J.E., McCammon, J.A. and Baker, N.A. (2004) PDB2PQR: an automated pipeline for the setup of Poisson–Boltzmann electrostatics calculations. *Nucleic Acids Res.*, **32**, W665-W667.

18. Curuksu, J., Zakrzewska, K. and Zacharias, M. (2008) Magnitude and direction of DNA bending induced by screw-axis orientation: influence of sequence, mismatches and abasic sites. *Nucleic Acids Res.*, **36**, 2268-2283.
